# Unveiling the potential biochemical effects of selected heterocyclic compounds as human Type-A γ-aminobutyric acid (GABA A) Modulator: An *Insilico* Approach

**DOI:** 10.1016/j.btre.2025.e00894

**Published:** 2025-04-11

**Authors:** Abel Kolawole Oyebamiji, Sunday Adewale Akintelu, Oluwakemi Ebenezer, Faith Eniola Olujinmi, David O. Adekunle, Adesoji Alani Olanrewaju, Omowumi Temitayo Akinola, Samson Olusegun Afolabi, Ehimen Anastasia Erazua, Ayodeji Arnold Olaseinde

**Affiliations:** a Department of Industrial Chemistry, University of Ilesa, Ilesa, Osun State, Nigeria; b Department of Physics, University of Alberta, Edmonton, AB, T6G 2E1, Canada; c Industrial Chemistry Programme, PMB 284, Iwo, Osun State, Nigeria; d Microbiology Programme, Bowen University, PMB 284, Iwo, Osun State, Nigeria; e Infochemistry Scientific Center, ITMO University, Saint Petersburg, 191002, Russia; f Department of Chemistry, University of Ibadan, Ibadan, Nigeria; g Department of Chemistry, Clemson University, Clemson, SC, USA; h Good Health and Wellbeing Research Clusters (SDG 03), University of Ilesa, Ilesa, Osun State, Nigeria

**Keywords:** Type-A γ-aminobutyric acid, DFT, molecular Modeling, scoring

## Abstract

**Background:**

Investigating the bioactivities of zuranolone derivatives as Type-A γ-aminobutyric acid inhibitors which will thereby down-regulate postpartum depression is considered a crucial study.

**Method:**

This study is aimed at investigating the biochemical activities of 1-(2-((3R,5R,8R,9R,10S,13S,14S,17S)-3‑hydroxy-3,13-dimethylhexadecahydro-1H-cyclopenta[a]phenanthren-17-yl)-2-oxoethyl)-1H-pyrazole-4-carbonitrile derivatives against type-A γ-aminobutyric acid (GABA A) which will thereby enhance the activity of γ-aminobutyric acid (GABA) in human central nervous system.

**Results:**

In this work, series of computational tools such as Spartan 14, molecular operating environment software, Gromacs and Admetsar1 were explored and the studied compounds were subjected to this software which resulted to series of results. Vacuum was observed to have highest influence on highest occupied molecular orbital (E_H_) of compound 1 and water as well as ethanol reduces its ability to donate electron to the nearby molecules. Also, the effect of water and ethanol were investigated on the studied compound via lowest unoccupied molecular orbital (E_L_) and energy gap and the results were reported appropriately. The molecular docking investigation was carried out on the studied compounds and Type-A γ-aminobutyric acid (pdb id: 4cof) and the compounds 3 with calculated binding affinity value of -7.32433319kcal/mol as well as pi-H as the non-bonding interaction were observed which therefore confirm the potential ability of compound to inhibit the target than other studied compound. Also, compound 1 and reference compound were subjected to molecular dynamic simulation study and the actual binding energy for the selected compounds were obtained and reported.

**Conclusion:**

Our findings from this work may open door for the design of several 1-(2-((3R,5R,8R,9R,10S,13S,14S,17S)-3-(benzyloxy)-3,13-dimethylhexadecahydro-1H-cyclopenta[a]phenanthren-17-yl)-2-oxoethyl)-1H-pyrazole-4-carbonitrile derivatives as potential Type-A γ-aminobutyric acid inhibitors.

## Highlights

• The antibacterial activity of Selected Heterocyclic compounds was evaluated.
• Descriptors found from optimized Selected Heterocyclic compounds were identified.
• Nonbonding interactions between drugs and the studied targets were observed.

## 1 Introduction

One of the challenging and strenuous practices that women experience in their life time is giving birth [21]. Several alterations such as hormonal, bodily, expressive and psychological changes are usually observed by women during pregnancy [6]. According to several scientists, remarkable vagaries take place in the female's marital and relational ecosphere [26,36] and series of variation in mother's behavior such as happiness, moodiness etc. could be observed after child birth and this is expected to fade off after fifteen days of giving birth [13].

Postpartum depression has been described by many researchers to be psychiatric related disease which happens to certain percentage (> 10 %) of women after child birth [34]. According to [4], perfect analysis of postpartum depression is challenging and this could be a function of resemblances in its demonstration with other attitude maladies. Several reports on postpartum depression in Africa have revealed different percentage of this menace in various regions in Africa [3,18,22]. The percentage rate of this disease in Democratic Republic of Congo and Cameroon, South Africa, Limbe, Nigeria and Uganda were 50.4 %, 23.4 %, 34.7 %, 61.8 %, 23.4 % and 6.6 % respectively [2,5,11]. Postpartum depression (PPD) is a serious mental health condition that affects many new mothers after childbirth. It can occur within the first few weeks to months following delivery and is characterized by feelings of extreme sadness, anxiety, and exhaustion [9,35]. Unlike the "baby blues," which are common and typically resolve within a few days, PPD can last longer and interfere with a mother's ability to care for herself and her baby. Symptoms may include difficulty bonding with the baby, feelings of hopelessness, irritability, sleep disturbances, and sometimes thoughts of self-harm or harming the baby. The causes of PPD are complex and can include hormonal changes, sleep deprivation, emotional stress, and a history of mental health issues. Seeking professional help is essential for managing PPD, and treatment often involves therapy, medication, or a combination of both to support recovery and well-being [8].

Human γ-aminobutyric acid (GABA) is recognized as a four-carbon amino acid and is found throughout the body [7]. Precious reports have shown that the various biological implications of GABA which could be found in living things have drawn the attention of many researchers [16]. According to [10] and [31], γ-aminobutyric acid is a key inhibitory neuro-transmitting agent in human nervous system which thereby has the ability to communicate info within human system, control neuronal growth and enhance sleep and frame of mind. Type-A γ-aminobutyric acid (GABA A) comprises of 5 (five) trans-membrane components which form a passage that is penetrable to chloride and the inflow of this chloride leads to destruction of neural action in human brain [28]. Therefore, effectively inhibiting type-A γ-aminobutyric acid (GABA-A) with a potent molecule to enhance its inhibitory activity is essential.

Moreover, heterocyclic compounds have been reported by various scientists to be efficient as inhibiting agents [14,15,20]. According to [24], the role played by heterocyclic compounds in drug design and development as potential inhibiting agent to several diseases such as cancer, diuretics and inflammation etc. have captured the attention of numerous researchers as well as production companies. Therefore, this study aims to investigate the biochemical activities of 1-(2-((3R,5R,8R,9R,10S,13S,14S,17S)-3‑hydroxy-3,13-dimethylhexadecahydro-1H-cyclopenta[a]phenanthren-17-yl)-2-oxoethyl)-1H-pyrazole-4-carbonitrile derivatives against Type-A γ-aminobutyric acid (GABA-A) using an in silico approach.

## 2 Methodology

### 2.1 Ligand minimization and optimization

The studied ligand were modeled using 3D style and optimized using 6–31+G* as basis set via Spartan 14 [29] (S1). The optimization of the ligands took place in vacuum, water and ethanol. In this calculation, equilibrium geometry at ground state was executed from current geometry together with neutral charge at zero (0) unpaired electron and the completion of the optimization of the studied ligands was observed to be a function of the atoms present in the compounds. The calculated features/descriptors were retrieved and presented accordingly. The IUPAC name of the 1-(2-((3R,5R,8R,9R,10S,13S,14S,17S)-3,13-dimethyl-3-((4-methylbenzyl)oxy)hexadecahydro-1H-cyclopenta[a]phenanthren-17-yl)-2-oxoethyl)-1H-pyrazole-4-carbonitrile (1), 1-(2-((3R,5R,8R,9R,10S,13S,14S,17S)-3-((4-chlorobenzyl)oxy)-3,13-dimethylhexadecahydro-1H-cyclopenta[a]phenanthren-17-yl)-2-oxoethyl)-1H-pyrazole-4-carbonitrile (2), 1-(2-((3R,5R,8R,9R,10S,13S,14S,17S)-3-(benzyloxy)-3,13-dimethylhexadecahydro-1H-cyclopenta[a]phenanthren-17-yl)-2-oxoethyl)-1H-pyrazole-4-carbonitrile (3), 1-(2-((3R,5R,8R,9R,10S,13S,14S,17S)-3‑methoxy-3,13-dimethylhexadecahydro-1H-cyclopenta[a]phenanthren-17-yl)-2-oxoethyl)-1H-pyrazole-4-carbonitrile (4), (3R,5R,8R,9R,10S,13S,14S,17S)-17-(2-(4-cyano-1H-pyrazol-1-yl)acetyl)-3,13-dimethylhexadecahydro-1H-cyclopenta[a]phenanthren-3-yl acetate (5), 1-(2-((3R,5R,8R,9R,10S,13S,14S,17S)-3,13-dimethyl-3-((4-nitrobenzyl)oxy)hexadecahydro-1H-cyclopenta[a]phenanthren-17-yl)-2-oxoethyl)-1H-pyrazole-4-carbonitrile (6), 1-(2-((3R,5R,8R,9R,10S,13S,14S,17S)-3-((4-bromobenzyl)oxy)-3,13-dimethylhexadecahydro-1H-cyclopenta[a]phenanthren-17-yl)-2-oxoethyl)-1H-pyrazole-4-carbonitrile (7), and 1-(2-((3R,5R,8R,9R,10S,13S,14S,17S)-3-((4-fluorobenzyl)oxy)-3,13-dimethylhexadecahydro-1H-cyclopenta[a]phenanthren-17-yl)-2-oxoethyl)-1H-pyrazole-4-carbonitrile (8).

### 2.2 Human Type-A γ-aminobutyric acid preparation

The retrieved receptor (Type-A γ-aminobutyric acid; pdb id: 4cof)) [19] from protein data bank was subjected quickPrep tool which comprises of appropriate sub tools for receptor preparations and saved in .moe format before docking calculation using molecular operating environment software. Also, the binding site was located using sitefinder and the suitable site was chosen for the docking calculation (Table 1). The treated and prepared protein structure was saved in .moe format before docking calculation using induced fit method. The obtained results from docked complexes were presented in kcal/mol and the types of interactions involved in the docked complexes were displayed and reported.

**Table 1:** Binding site components for Type-A γ-aminobutyric acid (pdb id: 4cof).

Site	Size	PLB	Hyd	Side	Residues
1	33	2.64	15	31	1:(PRO29 ASP30 VAL36 CYS37 TYR66 TRP67 ARG68 ASP69 LYS70 ARG71 ASP121 THR123 TYR167)
2	18	2.56	11	22	1:(PHE240 TRP241 ILE242 ASN243 TYR244 ARG250 ALA314 ASP424 ARG425 ARG428)
3	20	1.80	13	24	1:(GLU-2 THR-1 PHE11 GLU14 THR15 LYS18 LEU19 ALA73 TYR74 SER75 GLY76 ILE77)
4	11	0.35	9	14	1:(GLN224 THR225 PRO228 THR263 ILE264 HIS267 LEU268)
5	11	0.22	4	13	1:(CYS37 GLU165 PHE166 SER195 ARG196 ASN197 PRO206 ARG207)
6	17	0.18	17	25	1:(TYR97 LEU99 GLU155 SER156 TYR157 PHE200 THR202 TYR205)
7	13	0.06	6	12	1:(ASP139 LEU140 ARG141 ARG142 ASP146 GLU147 GLN148)
8	16	−0.08	12	18	1:(MET49 VAL50 SER51 LEU183 PRO184 GLN185 PHE186)
9	26	−0.15	11	17	1:(ARG142 PRO144 LEU145 ASP146 GLU147 LYS215 ARG216 ASN217 ILE218)
10	12	−0.28	6	8	1:(ARG269 GLU270 LEU272 PRO273 LYS274)
11	10	−0.34	13	17	1:(TYR97 PHE98 LEU99 ASP101 SER104 PHE105 VAL106 ILE130)
12	13	−0.36	12	16	1:(LYS13 VAL16 ASP17 LEU20 LEU83 ASP84 VAL87 GLN90)
13	6	−0.39	6	10	1:(ASP172 HIS191 ARG192 LEU193)
14	14	−0.42	8	14	1:(MET261 THR262 ASN265 THR266 ARG269 LEU285 MET286 PHE289)
15	3	−0.55	10	10	1:(LEU231 ILE232 ILE234 LEU235)
16	5	−0.56	8	11	1:(VAL251 TYR299 ALA300 ASN303 TYR304)
17	6	−0.57	5	7	1:(ASP17 LYS18 LEU20 LYS21)
18	4	−0.74	5	7	1:(SER436 ASN439 LEU440 TRP443)
19	7	−0.78	5	9	1:(GLU-2 THR-1 GLY0 GLN1 ASN4 PHE11)
20	5	−0.86	4	4	1:(PRO184 GLN185 ASN217 GLY219 TYR220)
21	5	−0.86	6	8	1:(TRP426 SER427 VAL430 PHE431)
22	6	−0.86	3	8	1:(MET55 MET137 MET138 PRO273 LYS274 ILE275 TYR277)

### 2.3 Molecular dynamic simulation analysis

Compound 4 and the Zuranolone-Type-A γ-aminobutyric acid complex were selected for molecular dynamic simulation studies due to their calculated high binding affinity, with Compound 4 and Zuranolone serving as the reference compound. These selected compounds were then processed using the SwissParam software (https://www.swissparam.ch/) for parameterization.

In this work, Charmm36m was employed as force field for the simulation of the parameterized compounds and the target using Gromacs software. Also, appropriate quantity of water molecules was added to the simulating system so as to accomplish solvation. More so, suitable ions were added at persistent pressure and temperature. The final simulation was executed via 100 nanoseconds and the obtained results were reported accordingly.

### 2.4 Calculation of pharmacokinetic properties of selected compounds

The features related to the Lipinski rule of five and other pharmacokinetic properties for Compound 4 and the reference compounds were analyzed and documented. ADMETSar 1 was used to perform this analysis, and the results were presented accordingly.

## 3 Results and discussion

### 3.1 Calculated density functional theory properties

The calculated descriptors obtained from the optimized compounds in vacuum, water and ethanol were reported in Table 2. The calculated descriptors for the optimized compounds were highest occupied molecular orbital energy (E_H_), lowest occupied molecular orbital energy (E_L_) and energy gap. The ability of the compound to release electron to compound close to it denote the strength of the compound to react greatly than other ligands; thus, 1-(2-((3R,5R,8R,9R,10S,13S,14S,17S)-3,13-dimethyl-3-((4-methylbenzyl)oxy)hexadecahydro-1H-cyclopenta[a]phenanthren-17-yl)-2-oxoethyl)-1H-pyrazole-4-carbonitrile (**1**) has E_H_ value of −6.47 eV and it denote that compound with highest strength to donate electron and this agreed with the report by [25]. More so, the report shown in Table 2 revealed the effect of solvents (water and ethanol) on the studied compounds. The calculated E_H_ for 1-(2-((3R,5R,8R,9R,10S,13S,14S,17S)-3,13-dimethyl-3-((4-methylbenzyl)oxy)hexadecahydro-1H-cyclopenta[a]phenanthren-17-yl)-2-oxoethyl)-1H-pyrazole-4-carbonitrile (**1**) were −6.47 eV, −6.63 eV and −6.62 eV in vacuum, water and ethanol respectively. It was observed that the solvents used in this work lower the donating strength of compound 1 and the order of its ability to donate electron were −6.47 eV (vacuum) > −6.62 eV (Ethanol) > −6.63 eV (Water).

**Table 2:** Calculated features for the studied compounds in vacuum, water and ethanol.

	Vacuum			Water			Ethanol		
	E_H_ (eV)	E_L_ (eV)	Energy Gap (eV)	E_H_ (eV)	E_L_ (eV)	Energy Gap (eV)	E_H_ (eV)	E_L_ (eV)	Energy Gap (eV)
1	−6.47	−1.74	4.73	−6.63	−1.66	4.97	−6.62	−1.63	4.99
2	−6.71	−1.77	4.94	−6.80	−1.68	5.12	−6.78	−1.65	5.13
3	−6.73	−1.74	4.99	−6.85	−1.65	5.20	−6.83	−1.62	5.21
4	−6.94	−1.76	5.18	−6.90	−1.71	5.19	−6.88	−1.67	5.21
5	−7.19	−1.81	5.38	−7.08	−1.66	5.42	−7.05	−1.63	5.42
6	−7.27	−3.68	3.59	−7.05	−4.02	3.03	−7.04	−3.94	3.10
7	−6.67	−1.76	4.91	−6.76	−1.67	5.09	−6.74	−1.64	5.10
8	−6.74	−1.76	4.98	−6.78	−1.66	5.12	−6.76	−1.63	5.13
ref	−7.13	−1.77	5.36	−7.08	−1.67	5.41	−7.05	−1.64	5.41

Furthermore, as shown in the order presented above, the connectivity between water and the atoms present in compound 1 proved that the reactivity of compound 1 became lowest when compared to the reactivity of compound 1 in gas phase and ethanol.

According to [30], ability to donate electron is not enough to justify the reactivity of any compound, therefore, the capability of any compound to receive electron is considered to be crucial [30]. Thus, 1-(2-((3R,5R,8R,9R,10S,13S,14S,17S)-3,13-dimethyl-3-((4-nitrobenzyl)oxy)hexadecahydro-1H-cyclopenta[a]phenanthren-17-yl)-2-oxoethyl)-1H-pyrazole-4-carbonitrile (6) was observed to be the compound with highest capacity to receive electron in all the phases studied in this work. This signifies that compound 6 has high tendency to react well than other studied compound. As shown in Table 2, it was observed that solvents used in this work has great effect on the receptive ability of compound **6** and the decreasing order of calculated E_L_ in all the three phases were −4.02 eV (Water) > −3.94 eV (Ethanol) > −3.68ev (Vacuum). According to the report presented in Table 2, it was detected that water enhanced the ability of compound 6 to receive electron more than other phases investigated in this work.

Furthermore, several scientists have reported that energy gap exposes the reactivity and stability indices of any molecule [1,27]. More so, spectroscopic properties of any examined complexes strongly depend on the calculated energy gap. The report by [17] revealed that lowest energy gap-based compounds have been observed to have propensity to be polarized easily and react well than other studied compounds. Therefore, as shown in Table 2, compound 6 could easily be polarized and showed a great tendency to react well with any possible neighboring compound. In this work, it could be observed that lowest unoccupied molecular orbital energy (E_L_) as well as compound 6 optimized in water supported the polarizability and high reacting strength of compound 6 which thereby resulted into lowest E_L_ values. This showed that water enhanced the reacting ability of compound 6 than other phases studied in this work.

### 3.2 Induced fit docking analysis

**Fig. 1:**
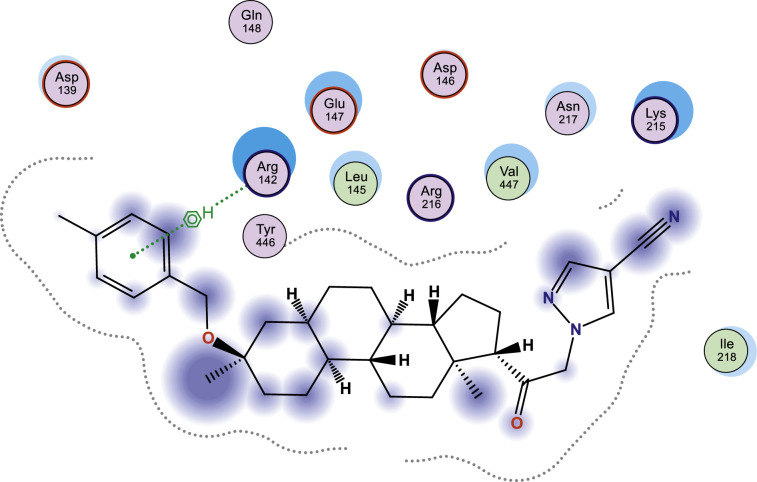
Two dimensional structure of compound 1 docked in Type-A γ-aminobutyric acid.

**Fig. 2:**
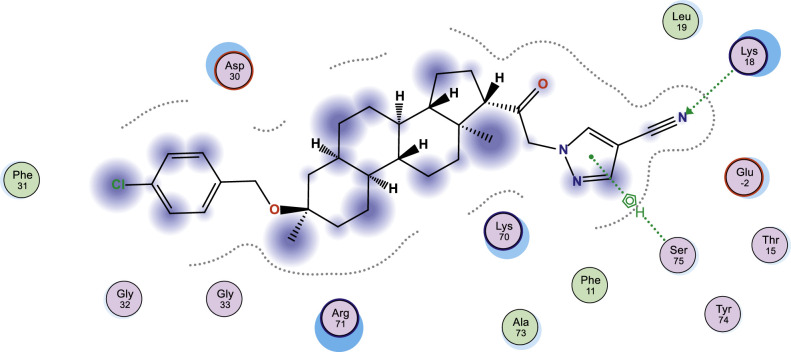
Two dimensional structure of compound 2 docked in Type-A γ-aminobutyric acid.

**Fig. 3:**
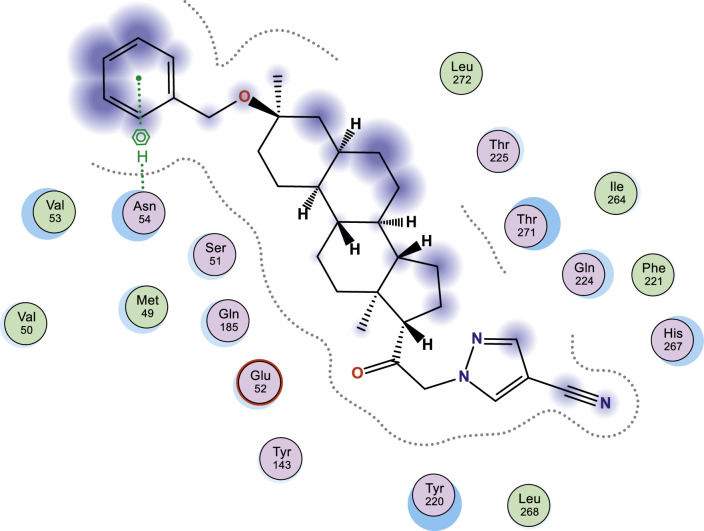
Two dimensional structure of compound 3 docked in Type-A γ-aminobutyric acid.

**Fig. 4:**
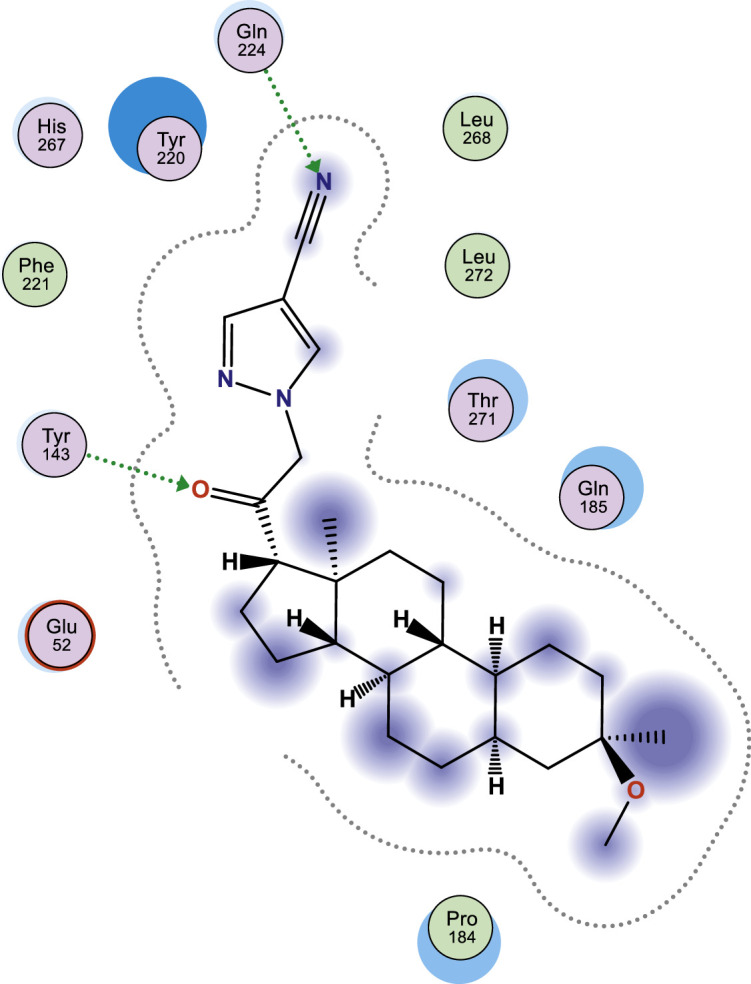
Two dimensional structure of compound 4 docked in Type-A γ-aminobutyric acid.

**Fig. 5:**
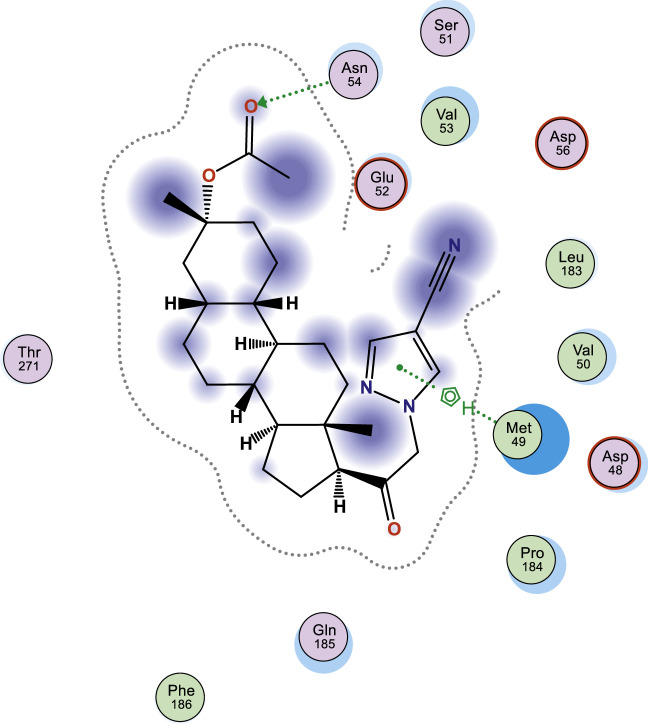
Two dimensional structure of compound 5 docked in Type-A γ-aminobutyric acid.

**Fig. 6:**
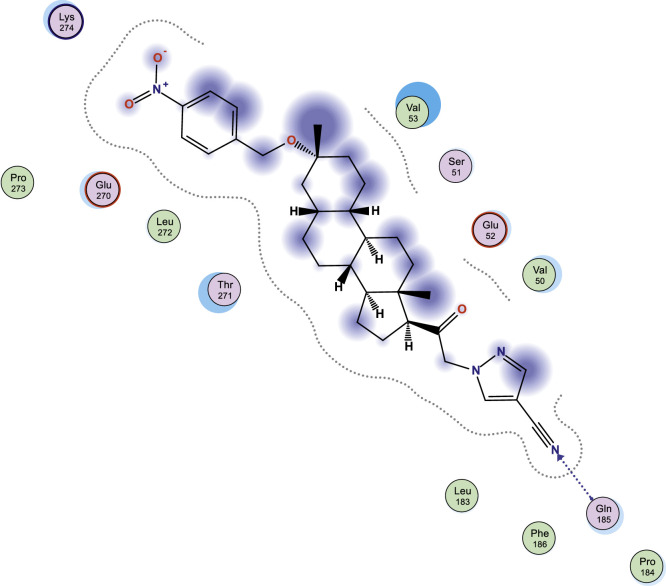
Two dimensional structure of compound 6 docked in Type-A γ-aminobutyric acid.

**Fig. 7:**
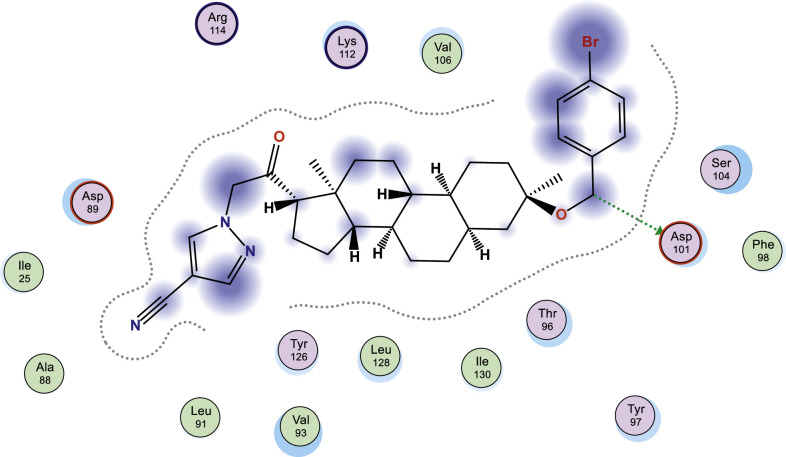
Two dimensional structure of compound 7 docked in Type-A γ-aminobutyric acid.

**Fig. 8:**
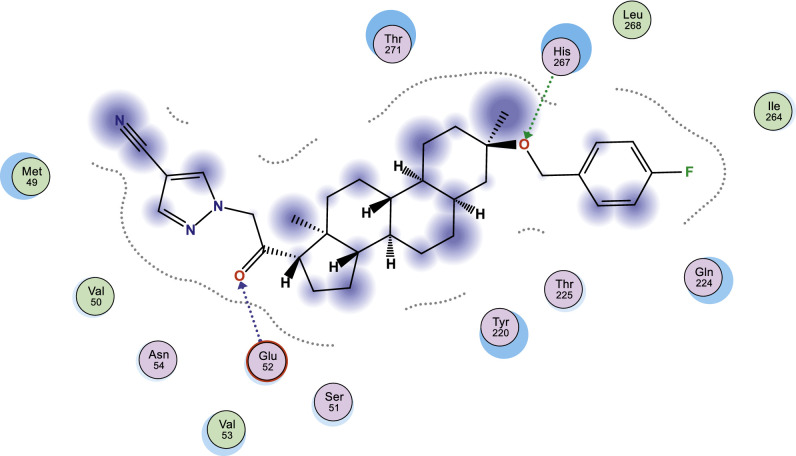
Two dimensional structure of compound 8 docked in Type-A γ-aminobutyric acid.

In this work, selected heterocyclic compounds were docked against Type-A γ-aminobutyric acid (GABA A) and were compared to inhibiting activities of Zuranolone against the target. As shown in Table 3, the activity of 1-(2-((3R,5R,8R,9R,10S,13S,14S,17S)-3-(benzyloxy)-3,13-dimethylhexadecahydro-1H-cyclopenta[a]phenanthren-17-yl)-2-oxoethyl)-1H-pyrazole-4-carbonitrile (**3**) was examined via the calculated binding affinity which was predicted to −7.32433319kcal/mol and as reported by [12], that lowest scoring value for any docked compound is considered to be the compound with highest binding affinity; thus, compound **3** is considered to be the ligand that has greatest strength to inhibit Type-A γ-aminobutyric acid (GABA A) than other studied ligands as well as Zuranolone (Reference ligand) (Fig. 1, Fig. 2, Fig. 3, Fig. 4, Fig. 5, Fig. 6, Fig. 7–8). The calculated scoring for each of studied ligands in kcal/mol, the part of the ligand involved in the interaction, the amino acid residues, the interaction and the distance calculated from the docked complex were presented in Table 3. As shown in this work, compound 1 – 3, 6 and 7 were observed to have greater efficiency to modulate the Type-A γ-aminobutyric acid (GABA A) which will thereby regulate postpartum depression.

**Table 3:** The calculated values for docking analysis.

	Scoring (kcal/mol)	Ligand	Receptor	Interaction	Distance
1	−7.28265476	6-ring	ARG 142	pi-H	3.63
2	−6.88447046	N 49 5-ring	LYS 18 SER 75	H-acceptor pi-H	3.45 4.16
3	−7.32433319	6-ring	ASN 54	pi-H	3.57
4	−6.10821772	N49 O50	GLN224 TYR143	H-acceptor H-acceptor	3.25 2.98
5	−6.03683615	O66 5-ring	ASN 54 MET 49	H-acceptor pi-H	2.87 4.12
6	−6.72247076	N49	GLN 185	H-acceptor	3.18
7	−6.79843283	C 65	ASP 101 (A)	H-donor	3.32
8	−6.38036156	O 50 O 64	GLU 52 HIS 267	H-acceptor H-acceptor	3.06 2.94
Ref	−6.38245916	–

### 3.3 Pharmacokinetics for compound 3 and reference compound

The studied pharmacokinetics for the selected compounds comprises of different classifications which are absorption distribution, metabolism and toxicity. The software used in this study did not return factor for excretion which was considered to be part of five classifications for pharmacokinetics study. As shown in Table 4, Table 5, the calculated values for human intestinal absorption for both selected compounds were observed to be 1 and it is an indication that the HIA is above the 30 % which therefore denotes moderate human intestinal absorption rate for the selected compounds [23].

**Table 4:** Predicted ADMET file for compound 3.

**ADMET Predicted Profile — Classification**		
**Model**	**Result**	**Probability**
**Absorption**		
Blood-Brain Barrier	BBB+	0.9771
Human Intestinal Absorption	HIA+	1.0000
Caco-2 Permeability	Caco2+	0.5189
P-glycoprotein Substrate	Substrate	0.5582
P-glycoprotein Inhibitor	Inhibitor	0.8777
	Inhibitor	0.9843
Renal Organic Cation Transporter	Inhibitor	0.6705
**Distribution**		
Subcellular localization	Mitochondria	0.6178
**Metabolism**		
CYP450 2C9 Substrate	Non-substrate	0.7781
CYP450 2D6 Substrate	Non-substrate	0.6454
CYP450 3A4 Substrate	Substrate	0.7356
CYP450 1A2 Inhibitor	Non-inhibitor	0.8866
CYP450 2C9 Inhibitor	Non-inhibitor	0.7513
CYP450 2D6 Inhibitor	Non-inhibitor	0.8644
CYP450 2C19 Inhibitor	Non-inhibitor	0.6012
CYP450 3A4 Inhibitor	Inhibitor	0.6242
CYP Inhibitory Promiscuity	Low CYP Inhibitory Promiscuity	0.5409
**Excretion**		
**Toxicity**		
Human Ether-a-go-go-Related Gene Inhibition	Weak inhibitor	0.7214
	Non-inhibitor	0.5131
AMES Toxicity	Non AMES toxic	0.6148
Carcinogens	Non-carcinogens	0.8689
Fish Toxicity	High FHMT	0.9965
Tetrahymena Pyriformis Toxicity	High TPT	0.9652
Honey Bee Toxicity	Low HBT	0.7038
Biodegradation	Not ready biodegradable	1.0000
Acute Oral Toxicity	III	0.6727
Carcinogenicity (Three-class)	Non-required	0.5316

**Table 5:** Predicted ADMET file for the studied reference compound.

**sModel**	**Result**	**Probability**
Absorption		
Blood-Brain Barrier	BBB+	0.9319
Human Intestinal Absorption	HIA+	1.0000
Caco-2 Permeability	Caco2+	0.5103
P-glycoprotein Substrate	Substrate	0.6073
P-glycoprotein Inhibitor	Inhibitor	0.7265
	Inhibitor	0.9491
Renal Organic Cation Transporter	Non-inhibitor	0.5177
**Distribution**		
Subcellular localization	Mitochondria	0.6570
**Metabolism**		
CYP450 2C9 Substrate	Non-substrate	0.7109
CYP450 2D6 Substrate	Non-substrate	0.6817
CYP450 3A4 Substrate	Substrate	0.7429
CYP450 1A2 Inhibitor	Non-inhibitor	0.8879
CYP450 2C9 Inhibitor	Non-inhibitor	0.7906
CYP450 2D6 Inhibitor	Non-inhibitor	0.8424
CYP450 2C19 Inhibitor	Non-inhibitor	0.6243
CYP450 3A4 Inhibitor	Inhibitor	0.6785
CYP Inhibitory Promiscuity	Low CYP Inhibitory Promiscuity	0.7850
**Excretion**		
**Toxicity**		
Human Ether-a-go-go-Related Gene Inhibition	Weak inhibitor	0.8462
	Non-inhibitor	0.7026
AMES Toxicity	Non AMES toxic	0.6850
Carcinogens	Non-carcinogens	0.8868
Fish Toxicity	High FHMT	0.9961
Tetrahymena Pyriformis Toxicity	High TPT	0.9749
Honey Bee Toxicity	Low HBT	0.7281
Biodegradation	Not ready biodegradable	1.0000
Acute Oral Toxicity	III	0.6401
Carcinogenicity (Three-class)	Non-required	0.5816

Also, as described by series of researchers, any compound could be considered appropriate based on the predicted blood brain barrier if the calculated value for BBB is ≥ 0.3; thus, the studied selected compounds were considered to be better in term of blood brain barrier as shown in supplementary Table 1 and 2 [33]. The predicted value for the selected compounds (0.6178 and 0.6570) for subcellular localization which described the distribution within human system prove to be good and they were observed to be higher than the referenced compound which agreed with the result reported by [32]. Other factors were obtained and reported accordingly.

## 4 Conclusion

In this study, eight heterocyclic compounds as human Type-A γ-aminobutyric acid (GABA-A) inhibitors were analyzed using computational methods. The interaction potential of these compounds was investigated through density functional theory by examining the calculated descriptors. Additionally, molecular modeling studies were conducted to evaluate the interaction between the compounds and GABA-A, and a series of binding affinities were reported. The results showed that 1-(2-((3R,5R,8R,9R,10S,13S,14S,17S)-3-(benzyloxy)-3,13-dimethylhexadecahydro-1H-cyclopenta[a]phenanthren-17-yl)-2-oxoethyl)-1H-pyrazole-4-carbonitrile (3) exhibited the highest binding affinity of −7.32433319 kcal/mol, indicating its strongest potential to inhibit the target. The predicted ADMET properties for compound 3, along with the reference compound, were also reported.

## Declarations

### Source of funding

Not Applicable

### Ethical approval

Not Applicable

## CRediT authorship contribution statement

**Abel Kolawole Oyebamiji:** Writing – review & editing, Writing – original draft, Formal analysis, Conceptualization. **Sunday Adewale Akintelu:** Supervision, Software, Resources, Project administration. **Oluwakemi Ebenezer:** Writing – original draft, Visualization, Formal analysis. **Faith Eniola Olujinmi:** Writing – original draft, Supervision, Resources. **David O. Adekunle:** Writing – original draft, Visualization, Resources, Investigation. **Adesoji Alani Olanrewaju:** Writing – original draft, Supervision, Software, Methodology. **Omowumi Temitayo Akinola:** Writing – original draft, Validation, Software, Conceptualization. **Samson Olusegun Afolabi:** Writing – original draft, Visualization, Validation, Supervision, Software, Conceptualization. **Ehimen Anastasia Erazua:** Visualization, Supervision, Project administration, Funding acquisition. **Ayodeji Arnold Olaseinde:** Writing – original draft, Resources, Project administration.

## Declaration of competing interest

The authors declare that they have no known competing financial interests or personal relationships that could have appeared to influence the work reported in this paper.

## Data Availability

All data were presented in the manuscript

## Appendix Supplementary materials

Image, application 1
