# Supplementary material for: Unveiling the potential biochemical effects of selected heterocyclic compounds as human Type-A γ-aminobutyric acid (GABA A) Modulator: An Insilico Approach
Source: Biotechnol Rep (Amst). 2025 Apr 11;46:e00894. doi: 10.1016/j.btre.2025.e00894 (PMC12032875; doi:10.1016/j.btre.2025.e00894)
Supplement: Supplementary file 1 [file mmc1.docx]

Supp Table 1:

**ADMET Predicted Profile --- Classification**

| **Model** | **Result** | **Probability** |
| --- | --- | --- |
| **Absorption** | | |
| Blood-Brain Barrier | [BBB+](http://lmmd.ecust.edu.cn/admetsar1/predict/?smiles=%5BH%5Dc1nn%28c%28%5BH%5D%29c1C%23N%29C%28%5BH%5D%29%28%5BH%5D%29C%28%3DO%29%5BC%40%40%5D1%28%5BH%5D%29C%28%5BH%5D%29%28%5BH%5D%29C%28%5BH%5D%29%28%5BH%5D%29%5BC%40%40%5D2%28%5BH%5D%29%5BC%40%5D3%28%5BH%5D%29C%28%5BH%5D%29%28%5BH%5D%29C%28%5BH%5D%29%28%5BH%5D%29%5BC%40%5D4%28%5BH%5D%29C%28%5BH%5D%29%28%5BH%5D%29%5BC%40%40%5D%28OC%28%5BH%5D%29%28%5BH%5D%29c5c%28%5BH%5D%29c%28%5BH%5D%29c%28%5BH%5D%29c%28%5BH%5D%29c5%5BH%5D%29%28C%28%5BH%5D%29%28%5BH%5D%29%5BH%5D%29C%28%5BH%5D%29%28%5BH%5D%29C%28%5BH%5D%29%28%5BH%5D%29%5BC%40%5D4%28%5BH%5D%29%5BC%40%40%5D3%28%5BH%5D%29C%28%5BH%5D%29%28%5BH%5D%29C%28%5BH%5D%29%28%5BH%5D%29%5BC%40%5D12C%28%5BH%5D%29%28%5BH%5D%29%5BH%5D+&action=A) | 0.9771 |
| Human Intestinal Absorption | [HIA+](http://lmmd.ecust.edu.cn/admetsar1/predict/?smiles=%5BH%5Dc1nn%28c%28%5BH%5D%29c1C%23N%29C%28%5BH%5D%29%28%5BH%5D%29C%28%3DO%29%5BC%40%40%5D1%28%5BH%5D%29C%28%5BH%5D%29%28%5BH%5D%29C%28%5BH%5D%29%28%5BH%5D%29%5BC%40%40%5D2%28%5BH%5D%29%5BC%40%5D3%28%5BH%5D%29C%28%5BH%5D%29%28%5BH%5D%29C%28%5BH%5D%29%28%5BH%5D%29%5BC%40%5D4%28%5BH%5D%29C%28%5BH%5D%29%28%5BH%5D%29%5BC%40%40%5D%28OC%28%5BH%5D%29%28%5BH%5D%29c5c%28%5BH%5D%29c%28%5BH%5D%29c%28%5BH%5D%29c%28%5BH%5D%29c5%5BH%5D%29%28C%28%5BH%5D%29%28%5BH%5D%29%5BH%5D%29C%28%5BH%5D%29%28%5BH%5D%29C%28%5BH%5D%29%28%5BH%5D%29%5BC%40%5D4%28%5BH%5D%29%5BC%40%40%5D3%28%5BH%5D%29C%28%5BH%5D%29%28%5BH%5D%29C%28%5BH%5D%29%28%5BH%5D%29%5BC%40%5D12C%28%5BH%5D%29%28%5BH%5D%29%5BH%5D+&action=A) | 1.0000 |
| Caco-2 Permeability | [Caco2+](http://lmmd.ecust.edu.cn/admetsar1/predict/?smiles=%5BH%5Dc1nn%28c%28%5BH%5D%29c1C%23N%29C%28%5BH%5D%29%28%5BH%5D%29C%28%3DO%29%5BC%40%40%5D1%28%5BH%5D%29C%28%5BH%5D%29%28%5BH%5D%29C%28%5BH%5D%29%28%5BH%5D%29%5BC%40%40%5D2%28%5BH%5D%29%5BC%40%5D3%28%5BH%5D%29C%28%5BH%5D%29%28%5BH%5D%29C%28%5BH%5D%29%28%5BH%5D%29%5BC%40%5D4%28%5BH%5D%29C%28%5BH%5D%29%28%5BH%5D%29%5BC%40%40%5D%28OC%28%5BH%5D%29%28%5BH%5D%29c5c%28%5BH%5D%29c%28%5BH%5D%29c%28%5BH%5D%29c%28%5BH%5D%29c5%5BH%5D%29%28C%28%5BH%5D%29%28%5BH%5D%29%5BH%5D%29C%28%5BH%5D%29%28%5BH%5D%29C%28%5BH%5D%29%28%5BH%5D%29%5BC%40%5D4%28%5BH%5D%29%5BC%40%40%5D3%28%5BH%5D%29C%28%5BH%5D%29%28%5BH%5D%29C%28%5BH%5D%29%28%5BH%5D%29%5BC%40%5D12C%28%5BH%5D%29%28%5BH%5D%29%5BH%5D+&action=A) | 0.5189 |
| P-glycoprotein Substrate | [Substrate](http://lmmd.ecust.edu.cn/admetsar1/predict/?smiles=%5BH%5Dc1nn%28c%28%5BH%5D%29c1C%23N%29C%28%5BH%5D%29%28%5BH%5D%29C%28%3DO%29%5BC%40%40%5D1%28%5BH%5D%29C%28%5BH%5D%29%28%5BH%5D%29C%28%5BH%5D%29%28%5BH%5D%29%5BC%40%40%5D2%28%5BH%5D%29%5BC%40%5D3%28%5BH%5D%29C%28%5BH%5D%29%28%5BH%5D%29C%28%5BH%5D%29%28%5BH%5D%29%5BC%40%5D4%28%5BH%5D%29C%28%5BH%5D%29%28%5BH%5D%29%5BC%40%40%5D%28OC%28%5BH%5D%29%28%5BH%5D%29c5c%28%5BH%5D%29c%28%5BH%5D%29c%28%5BH%5D%29c%28%5BH%5D%29c5%5BH%5D%29%28C%28%5BH%5D%29%28%5BH%5D%29%5BH%5D%29C%28%5BH%5D%29%28%5BH%5D%29C%28%5BH%5D%29%28%5BH%5D%29%5BC%40%5D4%28%5BH%5D%29%5BC%40%40%5D3%28%5BH%5D%29C%28%5BH%5D%29%28%5BH%5D%29C%28%5BH%5D%29%28%5BH%5D%29%5BC%40%5D12C%28%5BH%5D%29%28%5BH%5D%29%5BH%5D+&action=A) | 0.5582 |
| P-glycoprotein Inhibitor | [Inhibitor](http://lmmd.ecust.edu.cn/admetsar1/predict/?smiles=%5BH%5Dc1nn%28c%28%5BH%5D%29c1C%23N%29C%28%5BH%5D%29%28%5BH%5D%29C%28%3DO%29%5BC%40%40%5D1%28%5BH%5D%29C%28%5BH%5D%29%28%5BH%5D%29C%28%5BH%5D%29%28%5BH%5D%29%5BC%40%40%5D2%28%5BH%5D%29%5BC%40%5D3%28%5BH%5D%29C%28%5BH%5D%29%28%5BH%5D%29C%28%5BH%5D%29%28%5BH%5D%29%5BC%40%5D4%28%5BH%5D%29C%28%5BH%5D%29%28%5BH%5D%29%5BC%40%40%5D%28OC%28%5BH%5D%29%28%5BH%5D%29c5c%28%5BH%5D%29c%28%5BH%5D%29c%28%5BH%5D%29c%28%5BH%5D%29c5%5BH%5D%29%28C%28%5BH%5D%29%28%5BH%5D%29%5BH%5D%29C%28%5BH%5D%29%28%5BH%5D%29C%28%5BH%5D%29%28%5BH%5D%29%5BC%40%5D4%28%5BH%5D%29%5BC%40%40%5D3%28%5BH%5D%29C%28%5BH%5D%29%28%5BH%5D%29C%28%5BH%5D%29%28%5BH%5D%29%5BC%40%5D12C%28%5BH%5D%29%28%5BH%5D%29%5BH%5D+&action=A) | 0.8777 |
|  | [Inhibitor](http://lmmd.ecust.edu.cn/admetsar1/predict/?smiles=%5BH%5Dc1nn%28c%28%5BH%5D%29c1C%23N%29C%28%5BH%5D%29%28%5BH%5D%29C%28%3DO%29%5BC%40%40%5D1%28%5BH%5D%29C%28%5BH%5D%29%28%5BH%5D%29C%28%5BH%5D%29%28%5BH%5D%29%5BC%40%40%5D2%28%5BH%5D%29%5BC%40%5D3%28%5BH%5D%29C%28%5BH%5D%29%28%5BH%5D%29C%28%5BH%5D%29%28%5BH%5D%29%5BC%40%5D4%28%5BH%5D%29C%28%5BH%5D%29%28%5BH%5D%29%5BC%40%40%5D%28OC%28%5BH%5D%29%28%5BH%5D%29c5c%28%5BH%5D%29c%28%5BH%5D%29c%28%5BH%5D%29c%28%5BH%5D%29c5%5BH%5D%29%28C%28%5BH%5D%29%28%5BH%5D%29%5BH%5D%29C%28%5BH%5D%29%28%5BH%5D%29C%28%5BH%5D%29%28%5BH%5D%29%5BC%40%5D4%28%5BH%5D%29%5BC%40%40%5D3%28%5BH%5D%29C%28%5BH%5D%29%28%5BH%5D%29C%28%5BH%5D%29%28%5BH%5D%29%5BC%40%5D12C%28%5BH%5D%29%28%5BH%5D%29%5BH%5D+&action=A) | 0.9843 |
| Renal Organic Cation Transporter | [Inhibitor](http://lmmd.ecust.edu.cn/admetsar1/predict/?smiles=%5BH%5Dc1nn%28c%28%5BH%5D%29c1C%23N%29C%28%5BH%5D%29%28%5BH%5D%29C%28%3DO%29%5BC%40%40%5D1%28%5BH%5D%29C%28%5BH%5D%29%28%5BH%5D%29C%28%5BH%5D%29%28%5BH%5D%29%5BC%40%40%5D2%28%5BH%5D%29%5BC%40%5D3%28%5BH%5D%29C%28%5BH%5D%29%28%5BH%5D%29C%28%5BH%5D%29%28%5BH%5D%29%5BC%40%5D4%28%5BH%5D%29C%28%5BH%5D%29%28%5BH%5D%29%5BC%40%40%5D%28OC%28%5BH%5D%29%28%5BH%5D%29c5c%28%5BH%5D%29c%28%5BH%5D%29c%28%5BH%5D%29c%28%5BH%5D%29c5%5BH%5D%29%28C%28%5BH%5D%29%28%5BH%5D%29%5BH%5D%29C%28%5BH%5D%29%28%5BH%5D%29C%28%5BH%5D%29%28%5BH%5D%29%5BC%40%5D4%28%5BH%5D%29%5BC%40%40%5D3%28%5BH%5D%29C%28%5BH%5D%29%28%5BH%5D%29C%28%5BH%5D%29%28%5BH%5D%29%5BC%40%5D12C%28%5BH%5D%29%28%5BH%5D%29%5BH%5D+&action=A) | 0.6705 |
| **Distribution** | | |
| Subcellular localization | [Mitochondria](http://lmmd.ecust.edu.cn/admetsar1/predict/?smiles=%5BH%5Dc1nn%28c%28%5BH%5D%29c1C%23N%29C%28%5BH%5D%29%28%5BH%5D%29C%28%3DO%29%5BC%40%40%5D1%28%5BH%5D%29C%28%5BH%5D%29%28%5BH%5D%29C%28%5BH%5D%29%28%5BH%5D%29%5BC%40%40%5D2%28%5BH%5D%29%5BC%40%5D3%28%5BH%5D%29C%28%5BH%5D%29%28%5BH%5D%29C%28%5BH%5D%29%28%5BH%5D%29%5BC%40%5D4%28%5BH%5D%29C%28%5BH%5D%29%28%5BH%5D%29%5BC%40%40%5D%28OC%28%5BH%5D%29%28%5BH%5D%29c5c%28%5BH%5D%29c%28%5BH%5D%29c%28%5BH%5D%29c%28%5BH%5D%29c5%5BH%5D%29%28C%28%5BH%5D%29%28%5BH%5D%29%5BH%5D%29C%28%5BH%5D%29%28%5BH%5D%29C%28%5BH%5D%29%28%5BH%5D%29%5BC%40%5D4%28%5BH%5D%29%5BC%40%40%5D3%28%5BH%5D%29C%28%5BH%5D%29%28%5BH%5D%29C%28%5BH%5D%29%28%5BH%5D%29%5BC%40%5D12C%28%5BH%5D%29%28%5BH%5D%29%5BH%5D+&action=A) | 0.6178 |
| **Metabolism** | | |
| CYP450 2C9 Substrate | [Non-substrate](http://lmmd.ecust.edu.cn/admetsar1/predict/?smiles=%5BH%5Dc1nn%28c%28%5BH%5D%29c1C%23N%29C%28%5BH%5D%29%28%5BH%5D%29C%28%3DO%29%5BC%40%40%5D1%28%5BH%5D%29C%28%5BH%5D%29%28%5BH%5D%29C%28%5BH%5D%29%28%5BH%5D%29%5BC%40%40%5D2%28%5BH%5D%29%5BC%40%5D3%28%5BH%5D%29C%28%5BH%5D%29%28%5BH%5D%29C%28%5BH%5D%29%28%5BH%5D%29%5BC%40%5D4%28%5BH%5D%29C%28%5BH%5D%29%28%5BH%5D%29%5BC%40%40%5D%28OC%28%5BH%5D%29%28%5BH%5D%29c5c%28%5BH%5D%29c%28%5BH%5D%29c%28%5BH%5D%29c%28%5BH%5D%29c5%5BH%5D%29%28C%28%5BH%5D%29%28%5BH%5D%29%5BH%5D%29C%28%5BH%5D%29%28%5BH%5D%29C%28%5BH%5D%29%28%5BH%5D%29%5BC%40%5D4%28%5BH%5D%29%5BC%40%40%5D3%28%5BH%5D%29C%28%5BH%5D%29%28%5BH%5D%29C%28%5BH%5D%29%28%5BH%5D%29%5BC%40%5D12C%28%5BH%5D%29%28%5BH%5D%29%5BH%5D+&action=A) | 0.7781 |
| CYP450 2D6 Substrate | [Non-substrate](http://lmmd.ecust.edu.cn/admetsar1/predict/?smiles=%5BH%5Dc1nn%28c%28%5BH%5D%29c1C%23N%29C%28%5BH%5D%29%28%5BH%5D%29C%28%3DO%29%5BC%40%40%5D1%28%5BH%5D%29C%28%5BH%5D%29%28%5BH%5D%29C%28%5BH%5D%29%28%5BH%5D%29%5BC%40%40%5D2%28%5BH%5D%29%5BC%40%5D3%28%5BH%5D%29C%28%5BH%5D%29%28%5BH%5D%29C%28%5BH%5D%29%28%5BH%5D%29%5BC%40%5D4%28%5BH%5D%29C%28%5BH%5D%29%28%5BH%5D%29%5BC%40%40%5D%28OC%28%5BH%5D%29%28%5BH%5D%29c5c%28%5BH%5D%29c%28%5BH%5D%29c%28%5BH%5D%29c%28%5BH%5D%29c5%5BH%5D%29%28C%28%5BH%5D%29%28%5BH%5D%29%5BH%5D%29C%28%5BH%5D%29%28%5BH%5D%29C%28%5BH%5D%29%28%5BH%5D%29%5BC%40%5D4%28%5BH%5D%29%5BC%40%40%5D3%28%5BH%5D%29C%28%5BH%5D%29%28%5BH%5D%29C%28%5BH%5D%29%28%5BH%5D%29%5BC%40%5D12C%28%5BH%5D%29%28%5BH%5D%29%5BH%5D+&action=A) | 0.6454 |
| CYP450 3A4 Substrate | [Substrate](http://lmmd.ecust.edu.cn/admetsar1/predict/?smiles=%5BH%5Dc1nn%28c%28%5BH%5D%29c1C%23N%29C%28%5BH%5D%29%28%5BH%5D%29C%28%3DO%29%5BC%40%40%5D1%28%5BH%5D%29C%28%5BH%5D%29%28%5BH%5D%29C%28%5BH%5D%29%28%5BH%5D%29%5BC%40%40%5D2%28%5BH%5D%29%5BC%40%5D3%28%5BH%5D%29C%28%5BH%5D%29%28%5BH%5D%29C%28%5BH%5D%29%28%5BH%5D%29%5BC%40%5D4%28%5BH%5D%29C%28%5BH%5D%29%28%5BH%5D%29%5BC%40%40%5D%28OC%28%5BH%5D%29%28%5BH%5D%29c5c%28%5BH%5D%29c%28%5BH%5D%29c%28%5BH%5D%29c%28%5BH%5D%29c5%5BH%5D%29%28C%28%5BH%5D%29%28%5BH%5D%29%5BH%5D%29C%28%5BH%5D%29%28%5BH%5D%29C%28%5BH%5D%29%28%5BH%5D%29%5BC%40%5D4%28%5BH%5D%29%5BC%40%40%5D3%28%5BH%5D%29C%28%5BH%5D%29%28%5BH%5D%29C%28%5BH%5D%29%28%5BH%5D%29%5BC%40%5D12C%28%5BH%5D%29%28%5BH%5D%29%5BH%5D+&action=A) | 0.7356 |
| CYP450 1A2 Inhibitor | [Non-inhibitor](http://lmmd.ecust.edu.cn/admetsar1/predict/?smiles=%5BH%5Dc1nn%28c%28%5BH%5D%29c1C%23N%29C%28%5BH%5D%29%28%5BH%5D%29C%28%3DO%29%5BC%40%40%5D1%28%5BH%5D%29C%28%5BH%5D%29%28%5BH%5D%29C%28%5BH%5D%29%28%5BH%5D%29%5BC%40%40%5D2%28%5BH%5D%29%5BC%40%5D3%28%5BH%5D%29C%28%5BH%5D%29%28%5BH%5D%29C%28%5BH%5D%29%28%5BH%5D%29%5BC%40%5D4%28%5BH%5D%29C%28%5BH%5D%29%28%5BH%5D%29%5BC%40%40%5D%28OC%28%5BH%5D%29%28%5BH%5D%29c5c%28%5BH%5D%29c%28%5BH%5D%29c%28%5BH%5D%29c%28%5BH%5D%29c5%5BH%5D%29%28C%28%5BH%5D%29%28%5BH%5D%29%5BH%5D%29C%28%5BH%5D%29%28%5BH%5D%29C%28%5BH%5D%29%28%5BH%5D%29%5BC%40%5D4%28%5BH%5D%29%5BC%40%40%5D3%28%5BH%5D%29C%28%5BH%5D%29%28%5BH%5D%29C%28%5BH%5D%29%28%5BH%5D%29%5BC%40%5D12C%28%5BH%5D%29%28%5BH%5D%29%5BH%5D+&action=A) | 0.8866 |
| CYP450 2C9 Inhibitor | [Non-inhibitor](http://lmmd.ecust.edu.cn/admetsar1/predict/?smiles=%5BH%5Dc1nn%28c%28%5BH%5D%29c1C%23N%29C%28%5BH%5D%29%28%5BH%5D%29C%28%3DO%29%5BC%40%40%5D1%28%5BH%5D%29C%28%5BH%5D%29%28%5BH%5D%29C%28%5BH%5D%29%28%5BH%5D%29%5BC%40%40%5D2%28%5BH%5D%29%5BC%40%5D3%28%5BH%5D%29C%28%5BH%5D%29%28%5BH%5D%29C%28%5BH%5D%29%28%5BH%5D%29%5BC%40%5D4%28%5BH%5D%29C%28%5BH%5D%29%28%5BH%5D%29%5BC%40%40%5D%28OC%28%5BH%5D%29%28%5BH%5D%29c5c%28%5BH%5D%29c%28%5BH%5D%29c%28%5BH%5D%29c%28%5BH%5D%29c5%5BH%5D%29%28C%28%5BH%5D%29%28%5BH%5D%29%5BH%5D%29C%28%5BH%5D%29%28%5BH%5D%29C%28%5BH%5D%29%28%5BH%5D%29%5BC%40%5D4%28%5BH%5D%29%5BC%40%40%5D3%28%5BH%5D%29C%28%5BH%5D%29%28%5BH%5D%29C%28%5BH%5D%29%28%5BH%5D%29%5BC%40%5D12C%28%5BH%5D%29%28%5BH%5D%29%5BH%5D+&action=A) | 0.7513 |
| CYP450 2D6 Inhibitor | [Non-inhibitor](http://lmmd.ecust.edu.cn/admetsar1/predict/?smiles=%5BH%5Dc1nn%28c%28%5BH%5D%29c1C%23N%29C%28%5BH%5D%29%28%5BH%5D%29C%28%3DO%29%5BC%40%40%5D1%28%5BH%5D%29C%28%5BH%5D%29%28%5BH%5D%29C%28%5BH%5D%29%28%5BH%5D%29%5BC%40%40%5D2%28%5BH%5D%29%5BC%40%5D3%28%5BH%5D%29C%28%5BH%5D%29%28%5BH%5D%29C%28%5BH%5D%29%28%5BH%5D%29%5BC%40%5D4%28%5BH%5D%29C%28%5BH%5D%29%28%5BH%5D%29%5BC%40%40%5D%28OC%28%5BH%5D%29%28%5BH%5D%29c5c%28%5BH%5D%29c%28%5BH%5D%29c%28%5BH%5D%29c%28%5BH%5D%29c5%5BH%5D%29%28C%28%5BH%5D%29%28%5BH%5D%29%5BH%5D%29C%28%5BH%5D%29%28%5BH%5D%29C%28%5BH%5D%29%28%5BH%5D%29%5BC%40%5D4%28%5BH%5D%29%5BC%40%40%5D3%28%5BH%5D%29C%28%5BH%5D%29%28%5BH%5D%29C%28%5BH%5D%29%28%5BH%5D%29%5BC%40%5D12C%28%5BH%5D%29%28%5BH%5D%29%5BH%5D+&action=A) | 0.8644 |
| CYP450 2C19 Inhibitor | [Non-inhibitor](http://lmmd.ecust.edu.cn/admetsar1/predict/?smiles=%5BH%5Dc1nn%28c%28%5BH%5D%29c1C%23N%29C%28%5BH%5D%29%28%5BH%5D%29C%28%3DO%29%5BC%40%40%5D1%28%5BH%5D%29C%28%5BH%5D%29%28%5BH%5D%29C%28%5BH%5D%29%28%5BH%5D%29%5BC%40%40%5D2%28%5BH%5D%29%5BC%40%5D3%28%5BH%5D%29C%28%5BH%5D%29%28%5BH%5D%29C%28%5BH%5D%29%28%5BH%5D%29%5BC%40%5D4%28%5BH%5D%29C%28%5BH%5D%29%28%5BH%5D%29%5BC%40%40%5D%28OC%28%5BH%5D%29%28%5BH%5D%29c5c%28%5BH%5D%29c%28%5BH%5D%29c%28%5BH%5D%29c%28%5BH%5D%29c5%5BH%5D%29%28C%28%5BH%5D%29%28%5BH%5D%29%5BH%5D%29C%28%5BH%5D%29%28%5BH%5D%29C%28%5BH%5D%29%28%5BH%5D%29%5BC%40%5D4%28%5BH%5D%29%5BC%40%40%5D3%28%5BH%5D%29C%28%5BH%5D%29%28%5BH%5D%29C%28%5BH%5D%29%28%5BH%5D%29%5BC%40%5D12C%28%5BH%5D%29%28%5BH%5D%29%5BH%5D+&action=A) | 0.6012 |
| CYP450 3A4 Inhibitor | [Inhibitor](http://lmmd.ecust.edu.cn/admetsar1/predict/?smiles=%5BH%5Dc1nn%28c%28%5BH%5D%29c1C%23N%29C%28%5BH%5D%29%28%5BH%5D%29C%28%3DO%29%5BC%40%40%5D1%28%5BH%5D%29C%28%5BH%5D%29%28%5BH%5D%29C%28%5BH%5D%29%28%5BH%5D%29%5BC%40%40%5D2%28%5BH%5D%29%5BC%40%5D3%28%5BH%5D%29C%28%5BH%5D%29%28%5BH%5D%29C%28%5BH%5D%29%28%5BH%5D%29%5BC%40%5D4%28%5BH%5D%29C%28%5BH%5D%29%28%5BH%5D%29%5BC%40%40%5D%28OC%28%5BH%5D%29%28%5BH%5D%29c5c%28%5BH%5D%29c%28%5BH%5D%29c%28%5BH%5D%29c%28%5BH%5D%29c5%5BH%5D%29%28C%28%5BH%5D%29%28%5BH%5D%29%5BH%5D%29C%28%5BH%5D%29%28%5BH%5D%29C%28%5BH%5D%29%28%5BH%5D%29%5BC%40%5D4%28%5BH%5D%29%5BC%40%40%5D3%28%5BH%5D%29C%28%5BH%5D%29%28%5BH%5D%29C%28%5BH%5D%29%28%5BH%5D%29%5BC%40%5D12C%28%5BH%5D%29%28%5BH%5D%29%5BH%5D+&action=A) | 0.6242 |
| CYP Inhibitory Promiscuity | [Low CYP Inhibitory Promiscuity](http://lmmd.ecust.edu.cn/admetsar1/predict/?smiles=%5BH%5Dc1nn%28c%28%5BH%5D%29c1C%23N%29C%28%5BH%5D%29%28%5BH%5D%29C%28%3DO%29%5BC%40%40%5D1%28%5BH%5D%29C%28%5BH%5D%29%28%5BH%5D%29C%28%5BH%5D%29%28%5BH%5D%29%5BC%40%40%5D2%28%5BH%5D%29%5BC%40%5D3%28%5BH%5D%29C%28%5BH%5D%29%28%5BH%5D%29C%28%5BH%5D%29%28%5BH%5D%29%5BC%40%5D4%28%5BH%5D%29C%28%5BH%5D%29%28%5BH%5D%29%5BC%40%40%5D%28OC%28%5BH%5D%29%28%5BH%5D%29c5c%28%5BH%5D%29c%28%5BH%5D%29c%28%5BH%5D%29c%28%5BH%5D%29c5%5BH%5D%29%28C%28%5BH%5D%29%28%5BH%5D%29%5BH%5D%29C%28%5BH%5D%29%28%5BH%5D%29C%28%5BH%5D%29%28%5BH%5D%29%5BC%40%5D4%28%5BH%5D%29%5BC%40%40%5D3%28%5BH%5D%29C%28%5BH%5D%29%28%5BH%5D%29C%28%5BH%5D%29%28%5BH%5D%29%5BC%40%5D12C%28%5BH%5D%29%28%5BH%5D%29%5BH%5D+&action=A) | 0.5409 |
| **Excretion** | | |
| **Toxicity** | | |
| Human Ether-a-go-go-Related Gene Inhibition | [Weak inhibitor](http://lmmd.ecust.edu.cn/admetsar1/predict/?smiles=%5BH%5Dc1nn%28c%28%5BH%5D%29c1C%23N%29C%28%5BH%5D%29%28%5BH%5D%29C%28%3DO%29%5BC%40%40%5D1%28%5BH%5D%29C%28%5BH%5D%29%28%5BH%5D%29C%28%5BH%5D%29%28%5BH%5D%29%5BC%40%40%5D2%28%5BH%5D%29%5BC%40%5D3%28%5BH%5D%29C%28%5BH%5D%29%28%5BH%5D%29C%28%5BH%5D%29%28%5BH%5D%29%5BC%40%5D4%28%5BH%5D%29C%28%5BH%5D%29%28%5BH%5D%29%5BC%40%40%5D%28OC%28%5BH%5D%29%28%5BH%5D%29c5c%28%5BH%5D%29c%28%5BH%5D%29c%28%5BH%5D%29c%28%5BH%5D%29c5%5BH%5D%29%28C%28%5BH%5D%29%28%5BH%5D%29%5BH%5D%29C%28%5BH%5D%29%28%5BH%5D%29C%28%5BH%5D%29%28%5BH%5D%29%5BC%40%5D4%28%5BH%5D%29%5BC%40%40%5D3%28%5BH%5D%29C%28%5BH%5D%29%28%5BH%5D%29C%28%5BH%5D%29%28%5BH%5D%29%5BC%40%5D12C%28%5BH%5D%29%28%5BH%5D%29%5BH%5D+&action=A) | 0.7214 |
|  | [Non-inhibitor](http://lmmd.ecust.edu.cn/admetsar1/predict/?smiles=%5BH%5Dc1nn%28c%28%5BH%5D%29c1C%23N%29C%28%5BH%5D%29%28%5BH%5D%29C%28%3DO%29%5BC%40%40%5D1%28%5BH%5D%29C%28%5BH%5D%29%28%5BH%5D%29C%28%5BH%5D%29%28%5BH%5D%29%5BC%40%40%5D2%28%5BH%5D%29%5BC%40%5D3%28%5BH%5D%29C%28%5BH%5D%29%28%5BH%5D%29C%28%5BH%5D%29%28%5BH%5D%29%5BC%40%5D4%28%5BH%5D%29C%28%5BH%5D%29%28%5BH%5D%29%5BC%40%40%5D%28OC%28%5BH%5D%29%28%5BH%5D%29c5c%28%5BH%5D%29c%28%5BH%5D%29c%28%5BH%5D%29c%28%5BH%5D%29c5%5BH%5D%29%28C%28%5BH%5D%29%28%5BH%5D%29%5BH%5D%29C%28%5BH%5D%29%28%5BH%5D%29C%28%5BH%5D%29%28%5BH%5D%29%5BC%40%5D4%28%5BH%5D%29%5BC%40%40%5D3%28%5BH%5D%29C%28%5BH%5D%29%28%5BH%5D%29C%28%5BH%5D%29%28%5BH%5D%29%5BC%40%5D12C%28%5BH%5D%29%28%5BH%5D%29%5BH%5D+&action=A) | 0.5131 |
| AMES Toxicity | [Non AMES toxic](http://lmmd.ecust.edu.cn/admetsar1/predict/?smiles=%5BH%5Dc1nn%28c%28%5BH%5D%29c1C%23N%29C%28%5BH%5D%29%28%5BH%5D%29C%28%3DO%29%5BC%40%40%5D1%28%5BH%5D%29C%28%5BH%5D%29%28%5BH%5D%29C%28%5BH%5D%29%28%5BH%5D%29%5BC%40%40%5D2%28%5BH%5D%29%5BC%40%5D3%28%5BH%5D%29C%28%5BH%5D%29%28%5BH%5D%29C%28%5BH%5D%29%28%5BH%5D%29%5BC%40%5D4%28%5BH%5D%29C%28%5BH%5D%29%28%5BH%5D%29%5BC%40%40%5D%28OC%28%5BH%5D%29%28%5BH%5D%29c5c%28%5BH%5D%29c%28%5BH%5D%29c%28%5BH%5D%29c%28%5BH%5D%29c5%5BH%5D%29%28C%28%5BH%5D%29%28%5BH%5D%29%5BH%5D%29C%28%5BH%5D%29%28%5BH%5D%29C%28%5BH%5D%29%28%5BH%5D%29%5BC%40%5D4%28%5BH%5D%29%5BC%40%40%5D3%28%5BH%5D%29C%28%5BH%5D%29%28%5BH%5D%29C%28%5BH%5D%29%28%5BH%5D%29%5BC%40%5D12C%28%5BH%5D%29%28%5BH%5D%29%5BH%5D+&action=A) | 0.6148 |
| Carcinogens | [Non-carcinogens](http://lmmd.ecust.edu.cn/admetsar1/predict/?smiles=%5BH%5Dc1nn%28c%28%5BH%5D%29c1C%23N%29C%28%5BH%5D%29%28%5BH%5D%29C%28%3DO%29%5BC%40%40%5D1%28%5BH%5D%29C%28%5BH%5D%29%28%5BH%5D%29C%28%5BH%5D%29%28%5BH%5D%29%5BC%40%40%5D2%28%5BH%5D%29%5BC%40%5D3%28%5BH%5D%29C%28%5BH%5D%29%28%5BH%5D%29C%28%5BH%5D%29%28%5BH%5D%29%5BC%40%5D4%28%5BH%5D%29C%28%5BH%5D%29%28%5BH%5D%29%5BC%40%40%5D%28OC%28%5BH%5D%29%28%5BH%5D%29c5c%28%5BH%5D%29c%28%5BH%5D%29c%28%5BH%5D%29c%28%5BH%5D%29c5%5BH%5D%29%28C%28%5BH%5D%29%28%5BH%5D%29%5BH%5D%29C%28%5BH%5D%29%28%5BH%5D%29C%28%5BH%5D%29%28%5BH%5D%29%5BC%40%5D4%28%5BH%5D%29%5BC%40%40%5D3%28%5BH%5D%29C%28%5BH%5D%29%28%5BH%5D%29C%28%5BH%5D%29%28%5BH%5D%29%5BC%40%5D12C%28%5BH%5D%29%28%5BH%5D%29%5BH%5D+&action=A) | 0.8689 |
| Fish Toxicity | [High FHMT](http://lmmd.ecust.edu.cn/admetsar1/predict/?smiles=%5BH%5Dc1nn%28c%28%5BH%5D%29c1C%23N%29C%28%5BH%5D%29%28%5BH%5D%29C%28%3DO%29%5BC%40%40%5D1%28%5BH%5D%29C%28%5BH%5D%29%28%5BH%5D%29C%28%5BH%5D%29%28%5BH%5D%29%5BC%40%40%5D2%28%5BH%5D%29%5BC%40%5D3%28%5BH%5D%29C%28%5BH%5D%29%28%5BH%5D%29C%28%5BH%5D%29%28%5BH%5D%29%5BC%40%5D4%28%5BH%5D%29C%28%5BH%5D%29%28%5BH%5D%29%5BC%40%40%5D%28OC%28%5BH%5D%29%28%5BH%5D%29c5c%28%5BH%5D%29c%28%5BH%5D%29c%28%5BH%5D%29c%28%5BH%5D%29c5%5BH%5D%29%28C%28%5BH%5D%29%28%5BH%5D%29%5BH%5D%29C%28%5BH%5D%29%28%5BH%5D%29C%28%5BH%5D%29%28%5BH%5D%29%5BC%40%5D4%28%5BH%5D%29%5BC%40%40%5D3%28%5BH%5D%29C%28%5BH%5D%29%28%5BH%5D%29C%28%5BH%5D%29%28%5BH%5D%29%5BC%40%5D12C%28%5BH%5D%29%28%5BH%5D%29%5BH%5D+&action=A) | 0.9965 |
| Tetrahymena Pyriformis Toxicity | [High TPT](http://lmmd.ecust.edu.cn/admetsar1/predict/?smiles=%5BH%5Dc1nn%28c%28%5BH%5D%29c1C%23N%29C%28%5BH%5D%29%28%5BH%5D%29C%28%3DO%29%5BC%40%40%5D1%28%5BH%5D%29C%28%5BH%5D%29%28%5BH%5D%29C%28%5BH%5D%29%28%5BH%5D%29%5BC%40%40%5D2%28%5BH%5D%29%5BC%40%5D3%28%5BH%5D%29C%28%5BH%5D%29%28%5BH%5D%29C%28%5BH%5D%29%28%5BH%5D%29%5BC%40%5D4%28%5BH%5D%29C%28%5BH%5D%29%28%5BH%5D%29%5BC%40%40%5D%28OC%28%5BH%5D%29%28%5BH%5D%29c5c%28%5BH%5D%29c%28%5BH%5D%29c%28%5BH%5D%29c%28%5BH%5D%29c5%5BH%5D%29%28C%28%5BH%5D%29%28%5BH%5D%29%5BH%5D%29C%28%5BH%5D%29%28%5BH%5D%29C%28%5BH%5D%29%28%5BH%5D%29%5BC%40%5D4%28%5BH%5D%29%5BC%40%40%5D3%28%5BH%5D%29C%28%5BH%5D%29%28%5BH%5D%29C%28%5BH%5D%29%28%5BH%5D%29%5BC%40%5D12C%28%5BH%5D%29%28%5BH%5D%29%5BH%5D+&action=A) | 0.9652 |
| Honey Bee Toxicity | [Low HBT](http://lmmd.ecust.edu.cn/admetsar1/predict/?smiles=%5BH%5Dc1nn%28c%28%5BH%5D%29c1C%23N%29C%28%5BH%5D%29%28%5BH%5D%29C%28%3DO%29%5BC%40%40%5D1%28%5BH%5D%29C%28%5BH%5D%29%28%5BH%5D%29C%28%5BH%5D%29%28%5BH%5D%29%5BC%40%40%5D2%28%5BH%5D%29%5BC%40%5D3%28%5BH%5D%29C%28%5BH%5D%29%28%5BH%5D%29C%28%5BH%5D%29%28%5BH%5D%29%5BC%40%5D4%28%5BH%5D%29C%28%5BH%5D%29%28%5BH%5D%29%5BC%40%40%5D%28OC%28%5BH%5D%29%28%5BH%5D%29c5c%28%5BH%5D%29c%28%5BH%5D%29c%28%5BH%5D%29c%28%5BH%5D%29c5%5BH%5D%29%28C%28%5BH%5D%29%28%5BH%5D%29%5BH%5D%29C%28%5BH%5D%29%28%5BH%5D%29C%28%5BH%5D%29%28%5BH%5D%29%5BC%40%5D4%28%5BH%5D%29%5BC%40%40%5D3%28%5BH%5D%29C%28%5BH%5D%29%28%5BH%5D%29C%28%5BH%5D%29%28%5BH%5D%29%5BC%40%5D12C%28%5BH%5D%29%28%5BH%5D%29%5BH%5D+&action=A) | 0.7038 |
| Biodegradation | [Not ready biodegradable](http://lmmd.ecust.edu.cn/admetsar1/predict/?smiles=%5BH%5Dc1nn%28c%28%5BH%5D%29c1C%23N%29C%28%5BH%5D%29%28%5BH%5D%29C%28%3DO%29%5BC%40%40%5D1%28%5BH%5D%29C%28%5BH%5D%29%28%5BH%5D%29C%28%5BH%5D%29%28%5BH%5D%29%5BC%40%40%5D2%28%5BH%5D%29%5BC%40%5D3%28%5BH%5D%29C%28%5BH%5D%29%28%5BH%5D%29C%28%5BH%5D%29%28%5BH%5D%29%5BC%40%5D4%28%5BH%5D%29C%28%5BH%5D%29%28%5BH%5D%29%5BC%40%40%5D%28OC%28%5BH%5D%29%28%5BH%5D%29c5c%28%5BH%5D%29c%28%5BH%5D%29c%28%5BH%5D%29c%28%5BH%5D%29c5%5BH%5D%29%28C%28%5BH%5D%29%28%5BH%5D%29%5BH%5D%29C%28%5BH%5D%29%28%5BH%5D%29C%28%5BH%5D%29%28%5BH%5D%29%5BC%40%5D4%28%5BH%5D%29%5BC%40%40%5D3%28%5BH%5D%29C%28%5BH%5D%29%28%5BH%5D%29C%28%5BH%5D%29%28%5BH%5D%29%5BC%40%5D12C%28%5BH%5D%29%28%5BH%5D%29%5BH%5D+&action=A) | 1.0000 |
| Acute Oral Toxicity | [III](http://lmmd.ecust.edu.cn/admetsar1/predict/?smiles=%5BH%5Dc1nn%28c%28%5BH%5D%29c1C%23N%29C%28%5BH%5D%29%28%5BH%5D%29C%28%3DO%29%5BC%40%40%5D1%28%5BH%5D%29C%28%5BH%5D%29%28%5BH%5D%29C%28%5BH%5D%29%28%5BH%5D%29%5BC%40%40%5D2%28%5BH%5D%29%5BC%40%5D3%28%5BH%5D%29C%28%5BH%5D%29%28%5BH%5D%29C%28%5BH%5D%29%28%5BH%5D%29%5BC%40%5D4%28%5BH%5D%29C%28%5BH%5D%29%28%5BH%5D%29%5BC%40%40%5D%28OC%28%5BH%5D%29%28%5BH%5D%29c5c%28%5BH%5D%29c%28%5BH%5D%29c%28%5BH%5D%29c%28%5BH%5D%29c5%5BH%5D%29%28C%28%5BH%5D%29%28%5BH%5D%29%5BH%5D%29C%28%5BH%5D%29%28%5BH%5D%29C%28%5BH%5D%29%28%5BH%5D%29%5BC%40%5D4%28%5BH%5D%29%5BC%40%40%5D3%28%5BH%5D%29C%28%5BH%5D%29%28%5BH%5D%29C%28%5BH%5D%29%28%5BH%5D%29%5BC%40%5D12C%28%5BH%5D%29%28%5BH%5D%29%5BH%5D+&action=A) | 0.6727 |
| Carcinogenicity (Three-class) | [Non-required](http://lmmd.ecust.edu.cn/admetsar1/predict/?smiles=%5BH%5Dc1nn%28c%28%5BH%5D%29c1C%23N%29C%28%5BH%5D%29%28%5BH%5D%29C%28%3DO%29%5BC%40%40%5D1%28%5BH%5D%29C%28%5BH%5D%29%28%5BH%5D%29C%28%5BH%5D%29%28%5BH%5D%29%5BC%40%40%5D2%28%5BH%5D%29%5BC%40%5D3%28%5BH%5D%29C%28%5BH%5D%29%28%5BH%5D%29C%28%5BH%5D%29%28%5BH%5D%29%5BC%40%5D4%28%5BH%5D%29C%28%5BH%5D%29%28%5BH%5D%29%5BC%40%40%5D%28OC%28%5BH%5D%29%28%5BH%5D%29c5c%28%5BH%5D%29c%28%5BH%5D%29c%28%5BH%5D%29c%28%5BH%5D%29c5%5BH%5D%29%28C%28%5BH%5D%29%28%5BH%5D%29%5BH%5D%29C%28%5BH%5D%29%28%5BH%5D%29C%28%5BH%5D%29%28%5BH%5D%29%5BC%40%5D4%28%5BH%5D%29%5BC%40%40%5D3%28%5BH%5D%29C%28%5BH%5D%29%28%5BH%5D%29C%28%5BH%5D%29%28%5BH%5D%29%5BC%40%5D12C%28%5BH%5D%29%28%5BH%5D%29%5BH%5D+&action=A) | 0.5316 |

Supp Table 2:

| **sModel** | **Result** | **Probability** |
| --- | --- | --- |
| **Absorption** | | |
| Blood-Brain Barrier | [BBB+](http://lmmd.ecust.edu.cn/admetsar1/predict/?smiles=%5BH%5DO%5BC%40%5D1%28C%28%5BH%5D%29%28%5BH%5D%29%5BH%5D%29C%28%5BH%5D%29%28%5BH%5D%29C%28%5BH%5D%29%28%5BH%5D%29%5BC%40%40%5D2%28%5BH%5D%29%5BC%40%5D%28%5BH%5D%29%28C%28%5BH%5D%29%28%5BH%5D%29C%28%5BH%5D%29%28%5BH%5D%29%5BC%40%40%5D3%28%5BH%5D%29%5BC%40%5D4%28%5BH%5D%29C%28%5BH%5D%29%28%5BH%5D%29C%28%5BH%5D%29%28%5BH%5D%29%5BC%40%5D%28%5BH%5D%29%28C%28%3DO%29C%28%5BH%5D%29%28%5BH%5D%29n5nc%28%5BH%5D%29c%28C%23N%29c5%5BH%5D%29%5BC%40%40%5D4%28C%28%5BH%5D%29%28%5BH%5D%29%5BH%5D%29C%28%5BH%5D%29%28%5BH%5D%29C%28%5BH%5D%29%28%5BH%5D%29%5BC%40%5D23%5BH%5D%29C1%28%5BH%5D%29%5BH%5D+&action=A) | 0.9319 |
| Human Intestinal Absorption | [HIA+](http://lmmd.ecust.edu.cn/admetsar1/predict/?smiles=%5BH%5DO%5BC%40%5D1%28C%28%5BH%5D%29%28%5BH%5D%29%5BH%5D%29C%28%5BH%5D%29%28%5BH%5D%29C%28%5BH%5D%29%28%5BH%5D%29%5BC%40%40%5D2%28%5BH%5D%29%5BC%40%5D%28%5BH%5D%29%28C%28%5BH%5D%29%28%5BH%5D%29C%28%5BH%5D%29%28%5BH%5D%29%5BC%40%40%5D3%28%5BH%5D%29%5BC%40%5D4%28%5BH%5D%29C%28%5BH%5D%29%28%5BH%5D%29C%28%5BH%5D%29%28%5BH%5D%29%5BC%40%5D%28%5BH%5D%29%28C%28%3DO%29C%28%5BH%5D%29%28%5BH%5D%29n5nc%28%5BH%5D%29c%28C%23N%29c5%5BH%5D%29%5BC%40%40%5D4%28C%28%5BH%5D%29%28%5BH%5D%29%5BH%5D%29C%28%5BH%5D%29%28%5BH%5D%29C%28%5BH%5D%29%28%5BH%5D%29%5BC%40%5D23%5BH%5D%29C1%28%5BH%5D%29%5BH%5D+&action=A) | 1.0000 |
| Caco-2 Permeability | [Caco2+](http://lmmd.ecust.edu.cn/admetsar1/predict/?smiles=%5BH%5DO%5BC%40%5D1%28C%28%5BH%5D%29%28%5BH%5D%29%5BH%5D%29C%28%5BH%5D%29%28%5BH%5D%29C%28%5BH%5D%29%28%5BH%5D%29%5BC%40%40%5D2%28%5BH%5D%29%5BC%40%5D%28%5BH%5D%29%28C%28%5BH%5D%29%28%5BH%5D%29C%28%5BH%5D%29%28%5BH%5D%29%5BC%40%40%5D3%28%5BH%5D%29%5BC%40%5D4%28%5BH%5D%29C%28%5BH%5D%29%28%5BH%5D%29C%28%5BH%5D%29%28%5BH%5D%29%5BC%40%5D%28%5BH%5D%29%28C%28%3DO%29C%28%5BH%5D%29%28%5BH%5D%29n5nc%28%5BH%5D%29c%28C%23N%29c5%5BH%5D%29%5BC%40%40%5D4%28C%28%5BH%5D%29%28%5BH%5D%29%5BH%5D%29C%28%5BH%5D%29%28%5BH%5D%29C%28%5BH%5D%29%28%5BH%5D%29%5BC%40%5D23%5BH%5D%29C1%28%5BH%5D%29%5BH%5D+&action=A) | 0.5103 |
| P-glycoprotein Substrate | [Substrate](http://lmmd.ecust.edu.cn/admetsar1/predict/?smiles=%5BH%5DO%5BC%40%5D1%28C%28%5BH%5D%29%28%5BH%5D%29%5BH%5D%29C%28%5BH%5D%29%28%5BH%5D%29C%28%5BH%5D%29%28%5BH%5D%29%5BC%40%40%5D2%28%5BH%5D%29%5BC%40%5D%28%5BH%5D%29%28C%28%5BH%5D%29%28%5BH%5D%29C%28%5BH%5D%29%28%5BH%5D%29%5BC%40%40%5D3%28%5BH%5D%29%5BC%40%5D4%28%5BH%5D%29C%28%5BH%5D%29%28%5BH%5D%29C%28%5BH%5D%29%28%5BH%5D%29%5BC%40%5D%28%5BH%5D%29%28C%28%3DO%29C%28%5BH%5D%29%28%5BH%5D%29n5nc%28%5BH%5D%29c%28C%23N%29c5%5BH%5D%29%5BC%40%40%5D4%28C%28%5BH%5D%29%28%5BH%5D%29%5BH%5D%29C%28%5BH%5D%29%28%5BH%5D%29C%28%5BH%5D%29%28%5BH%5D%29%5BC%40%5D23%5BH%5D%29C1%28%5BH%5D%29%5BH%5D+&action=A) | 0.6073 |
| P-glycoprotein Inhibitor | [Inhibitor](http://lmmd.ecust.edu.cn/admetsar1/predict/?smiles=%5BH%5DO%5BC%40%5D1%28C%28%5BH%5D%29%28%5BH%5D%29%5BH%5D%29C%28%5BH%5D%29%28%5BH%5D%29C%28%5BH%5D%29%28%5BH%5D%29%5BC%40%40%5D2%28%5BH%5D%29%5BC%40%5D%28%5BH%5D%29%28C%28%5BH%5D%29%28%5BH%5D%29C%28%5BH%5D%29%28%5BH%5D%29%5BC%40%40%5D3%28%5BH%5D%29%5BC%40%5D4%28%5BH%5D%29C%28%5BH%5D%29%28%5BH%5D%29C%28%5BH%5D%29%28%5BH%5D%29%5BC%40%5D%28%5BH%5D%29%28C%28%3DO%29C%28%5BH%5D%29%28%5BH%5D%29n5nc%28%5BH%5D%29c%28C%23N%29c5%5BH%5D%29%5BC%40%40%5D4%28C%28%5BH%5D%29%28%5BH%5D%29%5BH%5D%29C%28%5BH%5D%29%28%5BH%5D%29C%28%5BH%5D%29%28%5BH%5D%29%5BC%40%5D23%5BH%5D%29C1%28%5BH%5D%29%5BH%5D+&action=A) | 0.7265 |
|  | [Inhibitor](http://lmmd.ecust.edu.cn/admetsar1/predict/?smiles=%5BH%5DO%5BC%40%5D1%28C%28%5BH%5D%29%28%5BH%5D%29%5BH%5D%29C%28%5BH%5D%29%28%5BH%5D%29C%28%5BH%5D%29%28%5BH%5D%29%5BC%40%40%5D2%28%5BH%5D%29%5BC%40%5D%28%5BH%5D%29%28C%28%5BH%5D%29%28%5BH%5D%29C%28%5BH%5D%29%28%5BH%5D%29%5BC%40%40%5D3%28%5BH%5D%29%5BC%40%5D4%28%5BH%5D%29C%28%5BH%5D%29%28%5BH%5D%29C%28%5BH%5D%29%28%5BH%5D%29%5BC%40%5D%28%5BH%5D%29%28C%28%3DO%29C%28%5BH%5D%29%28%5BH%5D%29n5nc%28%5BH%5D%29c%28C%23N%29c5%5BH%5D%29%5BC%40%40%5D4%28C%28%5BH%5D%29%28%5BH%5D%29%5BH%5D%29C%28%5BH%5D%29%28%5BH%5D%29C%28%5BH%5D%29%28%5BH%5D%29%5BC%40%5D23%5BH%5D%29C1%28%5BH%5D%29%5BH%5D+&action=A) | 0.9491 |
| Renal Organic Cation Transporter | [Non-inhibitor](http://lmmd.ecust.edu.cn/admetsar1/predict/?smiles=%5BH%5DO%5BC%40%5D1%28C%28%5BH%5D%29%28%5BH%5D%29%5BH%5D%29C%28%5BH%5D%29%28%5BH%5D%29C%28%5BH%5D%29%28%5BH%5D%29%5BC%40%40%5D2%28%5BH%5D%29%5BC%40%5D%28%5BH%5D%29%28C%28%5BH%5D%29%28%5BH%5D%29C%28%5BH%5D%29%28%5BH%5D%29%5BC%40%40%5D3%28%5BH%5D%29%5BC%40%5D4%28%5BH%5D%29C%28%5BH%5D%29%28%5BH%5D%29C%28%5BH%5D%29%28%5BH%5D%29%5BC%40%5D%28%5BH%5D%29%28C%28%3DO%29C%28%5BH%5D%29%28%5BH%5D%29n5nc%28%5BH%5D%29c%28C%23N%29c5%5BH%5D%29%5BC%40%40%5D4%28C%28%5BH%5D%29%28%5BH%5D%29%5BH%5D%29C%28%5BH%5D%29%28%5BH%5D%29C%28%5BH%5D%29%28%5BH%5D%29%5BC%40%5D23%5BH%5D%29C1%28%5BH%5D%29%5BH%5D+&action=A) | 0.5177 |
| **Distribution** | | |
| Subcellular localization | [Mitochondria](http://lmmd.ecust.edu.cn/admetsar1/predict/?smiles=%5BH%5DO%5BC%40%5D1%28C%28%5BH%5D%29%28%5BH%5D%29%5BH%5D%29C%28%5BH%5D%29%28%5BH%5D%29C%28%5BH%5D%29%28%5BH%5D%29%5BC%40%40%5D2%28%5BH%5D%29%5BC%40%5D%28%5BH%5D%29%28C%28%5BH%5D%29%28%5BH%5D%29C%28%5BH%5D%29%28%5BH%5D%29%5BC%40%40%5D3%28%5BH%5D%29%5BC%40%5D4%28%5BH%5D%29C%28%5BH%5D%29%28%5BH%5D%29C%28%5BH%5D%29%28%5BH%5D%29%5BC%40%5D%28%5BH%5D%29%28C%28%3DO%29C%28%5BH%5D%29%28%5BH%5D%29n5nc%28%5BH%5D%29c%28C%23N%29c5%5BH%5D%29%5BC%40%40%5D4%28C%28%5BH%5D%29%28%5BH%5D%29%5BH%5D%29C%28%5BH%5D%29%28%5BH%5D%29C%28%5BH%5D%29%28%5BH%5D%29%5BC%40%5D23%5BH%5D%29C1%28%5BH%5D%29%5BH%5D+&action=A) | 0.6570 |
| **Metabolism** | | |
| CYP450 2C9 Substrate | [Non-substrate](http://lmmd.ecust.edu.cn/admetsar1/predict/?smiles=%5BH%5DO%5BC%40%5D1%28C%28%5BH%5D%29%28%5BH%5D%29%5BH%5D%29C%28%5BH%5D%29%28%5BH%5D%29C%28%5BH%5D%29%28%5BH%5D%29%5BC%40%40%5D2%28%5BH%5D%29%5BC%40%5D%28%5BH%5D%29%28C%28%5BH%5D%29%28%5BH%5D%29C%28%5BH%5D%29%28%5BH%5D%29%5BC%40%40%5D3%28%5BH%5D%29%5BC%40%5D4%28%5BH%5D%29C%28%5BH%5D%29%28%5BH%5D%29C%28%5BH%5D%29%28%5BH%5D%29%5BC%40%5D%28%5BH%5D%29%28C%28%3DO%29C%28%5BH%5D%29%28%5BH%5D%29n5nc%28%5BH%5D%29c%28C%23N%29c5%5BH%5D%29%5BC%40%40%5D4%28C%28%5BH%5D%29%28%5BH%5D%29%5BH%5D%29C%28%5BH%5D%29%28%5BH%5D%29C%28%5BH%5D%29%28%5BH%5D%29%5BC%40%5D23%5BH%5D%29C1%28%5BH%5D%29%5BH%5D+&action=A) | 0.7109 |
| CYP450 2D6 Substrate | [Non-substrate](http://lmmd.ecust.edu.cn/admetsar1/predict/?smiles=%5BH%5DO%5BC%40%5D1%28C%28%5BH%5D%29%28%5BH%5D%29%5BH%5D%29C%28%5BH%5D%29%28%5BH%5D%29C%28%5BH%5D%29%28%5BH%5D%29%5BC%40%40%5D2%28%5BH%5D%29%5BC%40%5D%28%5BH%5D%29%28C%28%5BH%5D%29%28%5BH%5D%29C%28%5BH%5D%29%28%5BH%5D%29%5BC%40%40%5D3%28%5BH%5D%29%5BC%40%5D4%28%5BH%5D%29C%28%5BH%5D%29%28%5BH%5D%29C%28%5BH%5D%29%28%5BH%5D%29%5BC%40%5D%28%5BH%5D%29%28C%28%3DO%29C%28%5BH%5D%29%28%5BH%5D%29n5nc%28%5BH%5D%29c%28C%23N%29c5%5BH%5D%29%5BC%40%40%5D4%28C%28%5BH%5D%29%28%5BH%5D%29%5BH%5D%29C%28%5BH%5D%29%28%5BH%5D%29C%28%5BH%5D%29%28%5BH%5D%29%5BC%40%5D23%5BH%5D%29C1%28%5BH%5D%29%5BH%5D+&action=A) | 0.6817 |
| CYP450 3A4 Substrate | [Substrate](http://lmmd.ecust.edu.cn/admetsar1/predict/?smiles=%5BH%5DO%5BC%40%5D1%28C%28%5BH%5D%29%28%5BH%5D%29%5BH%5D%29C%28%5BH%5D%29%28%5BH%5D%29C%28%5BH%5D%29%28%5BH%5D%29%5BC%40%40%5D2%28%5BH%5D%29%5BC%40%5D%28%5BH%5D%29%28C%28%5BH%5D%29%28%5BH%5D%29C%28%5BH%5D%29%28%5BH%5D%29%5BC%40%40%5D3%28%5BH%5D%29%5BC%40%5D4%28%5BH%5D%29C%28%5BH%5D%29%28%5BH%5D%29C%28%5BH%5D%29%28%5BH%5D%29%5BC%40%5D%28%5BH%5D%29%28C%28%3DO%29C%28%5BH%5D%29%28%5BH%5D%29n5nc%28%5BH%5D%29c%28C%23N%29c5%5BH%5D%29%5BC%40%40%5D4%28C%28%5BH%5D%29%28%5BH%5D%29%5BH%5D%29C%28%5BH%5D%29%28%5BH%5D%29C%28%5BH%5D%29%28%5BH%5D%29%5BC%40%5D23%5BH%5D%29C1%28%5BH%5D%29%5BH%5D+&action=A) | 0.7429 |
| CYP450 1A2 Inhibitor | [Non-inhibitor](http://lmmd.ecust.edu.cn/admetsar1/predict/?smiles=%5BH%5DO%5BC%40%5D1%28C%28%5BH%5D%29%28%5BH%5D%29%5BH%5D%29C%28%5BH%5D%29%28%5BH%5D%29C%28%5BH%5D%29%28%5BH%5D%29%5BC%40%40%5D2%28%5BH%5D%29%5BC%40%5D%28%5BH%5D%29%28C%28%5BH%5D%29%28%5BH%5D%29C%28%5BH%5D%29%28%5BH%5D%29%5BC%40%40%5D3%28%5BH%5D%29%5BC%40%5D4%28%5BH%5D%29C%28%5BH%5D%29%28%5BH%5D%29C%28%5BH%5D%29%28%5BH%5D%29%5BC%40%5D%28%5BH%5D%29%28C%28%3DO%29C%28%5BH%5D%29%28%5BH%5D%29n5nc%28%5BH%5D%29c%28C%23N%29c5%5BH%5D%29%5BC%40%40%5D4%28C%28%5BH%5D%29%28%5BH%5D%29%5BH%5D%29C%28%5BH%5D%29%28%5BH%5D%29C%28%5BH%5D%29%28%5BH%5D%29%5BC%40%5D23%5BH%5D%29C1%28%5BH%5D%29%5BH%5D+&action=A) | 0.8879 |
| CYP450 2C9 Inhibitor | [Non-inhibitor](http://lmmd.ecust.edu.cn/admetsar1/predict/?smiles=%5BH%5DO%5BC%40%5D1%28C%28%5BH%5D%29%28%5BH%5D%29%5BH%5D%29C%28%5BH%5D%29%28%5BH%5D%29C%28%5BH%5D%29%28%5BH%5D%29%5BC%40%40%5D2%28%5BH%5D%29%5BC%40%5D%28%5BH%5D%29%28C%28%5BH%5D%29%28%5BH%5D%29C%28%5BH%5D%29%28%5BH%5D%29%5BC%40%40%5D3%28%5BH%5D%29%5BC%40%5D4%28%5BH%5D%29C%28%5BH%5D%29%28%5BH%5D%29C%28%5BH%5D%29%28%5BH%5D%29%5BC%40%5D%28%5BH%5D%29%28C%28%3DO%29C%28%5BH%5D%29%28%5BH%5D%29n5nc%28%5BH%5D%29c%28C%23N%29c5%5BH%5D%29%5BC%40%40%5D4%28C%28%5BH%5D%29%28%5BH%5D%29%5BH%5D%29C%28%5BH%5D%29%28%5BH%5D%29C%28%5BH%5D%29%28%5BH%5D%29%5BC%40%5D23%5BH%5D%29C1%28%5BH%5D%29%5BH%5D+&action=A) | 0.7906 |
| CYP450 2D6 Inhibitor | [Non-inhibitor](http://lmmd.ecust.edu.cn/admetsar1/predict/?smiles=%5BH%5DO%5BC%40%5D1%28C%28%5BH%5D%29%28%5BH%5D%29%5BH%5D%29C%28%5BH%5D%29%28%5BH%5D%29C%28%5BH%5D%29%28%5BH%5D%29%5BC%40%40%5D2%28%5BH%5D%29%5BC%40%5D%28%5BH%5D%29%28C%28%5BH%5D%29%28%5BH%5D%29C%28%5BH%5D%29%28%5BH%5D%29%5BC%40%40%5D3%28%5BH%5D%29%5BC%40%5D4%28%5BH%5D%29C%28%5BH%5D%29%28%5BH%5D%29C%28%5BH%5D%29%28%5BH%5D%29%5BC%40%5D%28%5BH%5D%29%28C%28%3DO%29C%28%5BH%5D%29%28%5BH%5D%29n5nc%28%5BH%5D%29c%28C%23N%29c5%5BH%5D%29%5BC%40%40%5D4%28C%28%5BH%5D%29%28%5BH%5D%29%5BH%5D%29C%28%5BH%5D%29%28%5BH%5D%29C%28%5BH%5D%29%28%5BH%5D%29%5BC%40%5D23%5BH%5D%29C1%28%5BH%5D%29%5BH%5D+&action=A) | 0.8424 |
| CYP450 2C19 Inhibitor | [Non-inhibitor](http://lmmd.ecust.edu.cn/admetsar1/predict/?smiles=%5BH%5DO%5BC%40%5D1%28C%28%5BH%5D%29%28%5BH%5D%29%5BH%5D%29C%28%5BH%5D%29%28%5BH%5D%29C%28%5BH%5D%29%28%5BH%5D%29%5BC%40%40%5D2%28%5BH%5D%29%5BC%40%5D%28%5BH%5D%29%28C%28%5BH%5D%29%28%5BH%5D%29C%28%5BH%5D%29%28%5BH%5D%29%5BC%40%40%5D3%28%5BH%5D%29%5BC%40%5D4%28%5BH%5D%29C%28%5BH%5D%29%28%5BH%5D%29C%28%5BH%5D%29%28%5BH%5D%29%5BC%40%5D%28%5BH%5D%29%28C%28%3DO%29C%28%5BH%5D%29%28%5BH%5D%29n5nc%28%5BH%5D%29c%28C%23N%29c5%5BH%5D%29%5BC%40%40%5D4%28C%28%5BH%5D%29%28%5BH%5D%29%5BH%5D%29C%28%5BH%5D%29%28%5BH%5D%29C%28%5BH%5D%29%28%5BH%5D%29%5BC%40%5D23%5BH%5D%29C1%28%5BH%5D%29%5BH%5D+&action=A) | 0.6243 |
| CYP450 3A4 Inhibitor | [Inhibitor](http://lmmd.ecust.edu.cn/admetsar1/predict/?smiles=%5BH%5DO%5BC%40%5D1%28C%28%5BH%5D%29%28%5BH%5D%29%5BH%5D%29C%28%5BH%5D%29%28%5BH%5D%29C%28%5BH%5D%29%28%5BH%5D%29%5BC%40%40%5D2%28%5BH%5D%29%5BC%40%5D%28%5BH%5D%29%28C%28%5BH%5D%29%28%5BH%5D%29C%28%5BH%5D%29%28%5BH%5D%29%5BC%40%40%5D3%28%5BH%5D%29%5BC%40%5D4%28%5BH%5D%29C%28%5BH%5D%29%28%5BH%5D%29C%28%5BH%5D%29%28%5BH%5D%29%5BC%40%5D%28%5BH%5D%29%28C%28%3DO%29C%28%5BH%5D%29%28%5BH%5D%29n5nc%28%5BH%5D%29c%28C%23N%29c5%5BH%5D%29%5BC%40%40%5D4%28C%28%5BH%5D%29%28%5BH%5D%29%5BH%5D%29C%28%5BH%5D%29%28%5BH%5D%29C%28%5BH%5D%29%28%5BH%5D%29%5BC%40%5D23%5BH%5D%29C1%28%5BH%5D%29%5BH%5D+&action=A) | 0.6785 |
| CYP Inhibitory Promiscuity | [Low CYP Inhibitory Promiscuity](http://lmmd.ecust.edu.cn/admetsar1/predict/?smiles=%5BH%5DO%5BC%40%5D1%28C%28%5BH%5D%29%28%5BH%5D%29%5BH%5D%29C%28%5BH%5D%29%28%5BH%5D%29C%28%5BH%5D%29%28%5BH%5D%29%5BC%40%40%5D2%28%5BH%5D%29%5BC%40%5D%28%5BH%5D%29%28C%28%5BH%5D%29%28%5BH%5D%29C%28%5BH%5D%29%28%5BH%5D%29%5BC%40%40%5D3%28%5BH%5D%29%5BC%40%5D4%28%5BH%5D%29C%28%5BH%5D%29%28%5BH%5D%29C%28%5BH%5D%29%28%5BH%5D%29%5BC%40%5D%28%5BH%5D%29%28C%28%3DO%29C%28%5BH%5D%29%28%5BH%5D%29n5nc%28%5BH%5D%29c%28C%23N%29c5%5BH%5D%29%5BC%40%40%5D4%28C%28%5BH%5D%29%28%5BH%5D%29%5BH%5D%29C%28%5BH%5D%29%28%5BH%5D%29C%28%5BH%5D%29%28%5BH%5D%29%5BC%40%5D23%5BH%5D%29C1%28%5BH%5D%29%5BH%5D+&action=A) | 0.7850 |
| **Excretion** | | |
| **Toxicity** | | |
| Human Ether-a-go-go-Related Gene Inhibition | [Weak inhibitor](http://lmmd.ecust.edu.cn/admetsar1/predict/?smiles=%5BH%5DO%5BC%40%5D1%28C%28%5BH%5D%29%28%5BH%5D%29%5BH%5D%29C%28%5BH%5D%29%28%5BH%5D%29C%28%5BH%5D%29%28%5BH%5D%29%5BC%40%40%5D2%28%5BH%5D%29%5BC%40%5D%28%5BH%5D%29%28C%28%5BH%5D%29%28%5BH%5D%29C%28%5BH%5D%29%28%5BH%5D%29%5BC%40%40%5D3%28%5BH%5D%29%5BC%40%5D4%28%5BH%5D%29C%28%5BH%5D%29%28%5BH%5D%29C%28%5BH%5D%29%28%5BH%5D%29%5BC%40%5D%28%5BH%5D%29%28C%28%3DO%29C%28%5BH%5D%29%28%5BH%5D%29n5nc%28%5BH%5D%29c%28C%23N%29c5%5BH%5D%29%5BC%40%40%5D4%28C%28%5BH%5D%29%28%5BH%5D%29%5BH%5D%29C%28%5BH%5D%29%28%5BH%5D%29C%28%5BH%5D%29%28%5BH%5D%29%5BC%40%5D23%5BH%5D%29C1%28%5BH%5D%29%5BH%5D+&action=A) | 0.8462 |
|  | [Non-inhibitor](http://lmmd.ecust.edu.cn/admetsar1/predict/?smiles=%5BH%5DO%5BC%40%5D1%28C%28%5BH%5D%29%28%5BH%5D%29%5BH%5D%29C%28%5BH%5D%29%28%5BH%5D%29C%28%5BH%5D%29%28%5BH%5D%29%5BC%40%40%5D2%28%5BH%5D%29%5BC%40%5D%28%5BH%5D%29%28C%28%5BH%5D%29%28%5BH%5D%29C%28%5BH%5D%29%28%5BH%5D%29%5BC%40%40%5D3%28%5BH%5D%29%5BC%40%5D4%28%5BH%5D%29C%28%5BH%5D%29%28%5BH%5D%29C%28%5BH%5D%29%28%5BH%5D%29%5BC%40%5D%28%5BH%5D%29%28C%28%3DO%29C%28%5BH%5D%29%28%5BH%5D%29n5nc%28%5BH%5D%29c%28C%23N%29c5%5BH%5D%29%5BC%40%40%5D4%28C%28%5BH%5D%29%28%5BH%5D%29%5BH%5D%29C%28%5BH%5D%29%28%5BH%5D%29C%28%5BH%5D%29%28%5BH%5D%29%5BC%40%5D23%5BH%5D%29C1%28%5BH%5D%29%5BH%5D+&action=A) | 0.7026 |
| AMES Toxicity | [Non AMES toxic](http://lmmd.ecust.edu.cn/admetsar1/predict/?smiles=%5BH%5DO%5BC%40%5D1%28C%28%5BH%5D%29%28%5BH%5D%29%5BH%5D%29C%28%5BH%5D%29%28%5BH%5D%29C%28%5BH%5D%29%28%5BH%5D%29%5BC%40%40%5D2%28%5BH%5D%29%5BC%40%5D%28%5BH%5D%29%28C%28%5BH%5D%29%28%5BH%5D%29C%28%5BH%5D%29%28%5BH%5D%29%5BC%40%40%5D3%28%5BH%5D%29%5BC%40%5D4%28%5BH%5D%29C%28%5BH%5D%29%28%5BH%5D%29C%28%5BH%5D%29%28%5BH%5D%29%5BC%40%5D%28%5BH%5D%29%28C%28%3DO%29C%28%5BH%5D%29%28%5BH%5D%29n5nc%28%5BH%5D%29c%28C%23N%29c5%5BH%5D%29%5BC%40%40%5D4%28C%28%5BH%5D%29%28%5BH%5D%29%5BH%5D%29C%28%5BH%5D%29%28%5BH%5D%29C%28%5BH%5D%29%28%5BH%5D%29%5BC%40%5D23%5BH%5D%29C1%28%5BH%5D%29%5BH%5D+&action=A) | 0.6850 |
| Carcinogens | [Non-carcinogens](http://lmmd.ecust.edu.cn/admetsar1/predict/?smiles=%5BH%5DO%5BC%40%5D1%28C%28%5BH%5D%29%28%5BH%5D%29%5BH%5D%29C%28%5BH%5D%29%28%5BH%5D%29C%28%5BH%5D%29%28%5BH%5D%29%5BC%40%40%5D2%28%5BH%5D%29%5BC%40%5D%28%5BH%5D%29%28C%28%5BH%5D%29%28%5BH%5D%29C%28%5BH%5D%29%28%5BH%5D%29%5BC%40%40%5D3%28%5BH%5D%29%5BC%40%5D4%28%5BH%5D%29C%28%5BH%5D%29%28%5BH%5D%29C%28%5BH%5D%29%28%5BH%5D%29%5BC%40%5D%28%5BH%5D%29%28C%28%3DO%29C%28%5BH%5D%29%28%5BH%5D%29n5nc%28%5BH%5D%29c%28C%23N%29c5%5BH%5D%29%5BC%40%40%5D4%28C%28%5BH%5D%29%28%5BH%5D%29%5BH%5D%29C%28%5BH%5D%29%28%5BH%5D%29C%28%5BH%5D%29%28%5BH%5D%29%5BC%40%5D23%5BH%5D%29C1%28%5BH%5D%29%5BH%5D+&action=A) | 0.8868 |
| Fish Toxicity | [High FHMT](http://lmmd.ecust.edu.cn/admetsar1/predict/?smiles=%5BH%5DO%5BC%40%5D1%28C%28%5BH%5D%29%28%5BH%5D%29%5BH%5D%29C%28%5BH%5D%29%28%5BH%5D%29C%28%5BH%5D%29%28%5BH%5D%29%5BC%40%40%5D2%28%5BH%5D%29%5BC%40%5D%28%5BH%5D%29%28C%28%5BH%5D%29%28%5BH%5D%29C%28%5BH%5D%29%28%5BH%5D%29%5BC%40%40%5D3%28%5BH%5D%29%5BC%40%5D4%28%5BH%5D%29C%28%5BH%5D%29%28%5BH%5D%29C%28%5BH%5D%29%28%5BH%5D%29%5BC%40%5D%28%5BH%5D%29%28C%28%3DO%29C%28%5BH%5D%29%28%5BH%5D%29n5nc%28%5BH%5D%29c%28C%23N%29c5%5BH%5D%29%5BC%40%40%5D4%28C%28%5BH%5D%29%28%5BH%5D%29%5BH%5D%29C%28%5BH%5D%29%28%5BH%5D%29C%28%5BH%5D%29%28%5BH%5D%29%5BC%40%5D23%5BH%5D%29C1%28%5BH%5D%29%5BH%5D+&action=A) | 0.9961 |
| Tetrahymena Pyriformis Toxicity | [High TPT](http://lmmd.ecust.edu.cn/admetsar1/predict/?smiles=%5BH%5DO%5BC%40%5D1%28C%28%5BH%5D%29%28%5BH%5D%29%5BH%5D%29C%28%5BH%5D%29%28%5BH%5D%29C%28%5BH%5D%29%28%5BH%5D%29%5BC%40%40%5D2%28%5BH%5D%29%5BC%40%5D%28%5BH%5D%29%28C%28%5BH%5D%29%28%5BH%5D%29C%28%5BH%5D%29%28%5BH%5D%29%5BC%40%40%5D3%28%5BH%5D%29%5BC%40%5D4%28%5BH%5D%29C%28%5BH%5D%29%28%5BH%5D%29C%28%5BH%5D%29%28%5BH%5D%29%5BC%40%5D%28%5BH%5D%29%28C%28%3DO%29C%28%5BH%5D%29%28%5BH%5D%29n5nc%28%5BH%5D%29c%28C%23N%29c5%5BH%5D%29%5BC%40%40%5D4%28C%28%5BH%5D%29%28%5BH%5D%29%5BH%5D%29C%28%5BH%5D%29%28%5BH%5D%29C%28%5BH%5D%29%28%5BH%5D%29%5BC%40%5D23%5BH%5D%29C1%28%5BH%5D%29%5BH%5D+&action=A) | 0.9749 |
| Honey Bee Toxicity | [Low HBT](http://lmmd.ecust.edu.cn/admetsar1/predict/?smiles=%5BH%5DO%5BC%40%5D1%28C%28%5BH%5D%29%28%5BH%5D%29%5BH%5D%29C%28%5BH%5D%29%28%5BH%5D%29C%28%5BH%5D%29%28%5BH%5D%29%5BC%40%40%5D2%28%5BH%5D%29%5BC%40%5D%28%5BH%5D%29%28C%28%5BH%5D%29%28%5BH%5D%29C%28%5BH%5D%29%28%5BH%5D%29%5BC%40%40%5D3%28%5BH%5D%29%5BC%40%5D4%28%5BH%5D%29C%28%5BH%5D%29%28%5BH%5D%29C%28%5BH%5D%29%28%5BH%5D%29%5BC%40%5D%28%5BH%5D%29%28C%28%3DO%29C%28%5BH%5D%29%28%5BH%5D%29n5nc%28%5BH%5D%29c%28C%23N%29c5%5BH%5D%29%5BC%40%40%5D4%28C%28%5BH%5D%29%28%5BH%5D%29%5BH%5D%29C%28%5BH%5D%29%28%5BH%5D%29C%28%5BH%5D%29%28%5BH%5D%29%5BC%40%5D23%5BH%5D%29C1%28%5BH%5D%29%5BH%5D+&action=A) | 0.7281 |
| Biodegradation | [Not ready biodegradable](http://lmmd.ecust.edu.cn/admetsar1/predict/?smiles=%5BH%5DO%5BC%40%5D1%28C%28%5BH%5D%29%28%5BH%5D%29%5BH%5D%29C%28%5BH%5D%29%28%5BH%5D%29C%28%5BH%5D%29%28%5BH%5D%29%5BC%40%40%5D2%28%5BH%5D%29%5BC%40%5D%28%5BH%5D%29%28C%28%5BH%5D%29%28%5BH%5D%29C%28%5BH%5D%29%28%5BH%5D%29%5BC%40%40%5D3%28%5BH%5D%29%5BC%40%5D4%28%5BH%5D%29C%28%5BH%5D%29%28%5BH%5D%29C%28%5BH%5D%29%28%5BH%5D%29%5BC%40%5D%28%5BH%5D%29%28C%28%3DO%29C%28%5BH%5D%29%28%5BH%5D%29n5nc%28%5BH%5D%29c%28C%23N%29c5%5BH%5D%29%5BC%40%40%5D4%28C%28%5BH%5D%29%28%5BH%5D%29%5BH%5D%29C%28%5BH%5D%29%28%5BH%5D%29C%28%5BH%5D%29%28%5BH%5D%29%5BC%40%5D23%5BH%5D%29C1%28%5BH%5D%29%5BH%5D+&action=A) | 1.0000 |
| Acute Oral Toxicity | [III](http://lmmd.ecust.edu.cn/admetsar1/predict/?smiles=%5BH%5DO%5BC%40%5D1%28C%28%5BH%5D%29%28%5BH%5D%29%5BH%5D%29C%28%5BH%5D%29%28%5BH%5D%29C%28%5BH%5D%29%28%5BH%5D%29%5BC%40%40%5D2%28%5BH%5D%29%5BC%40%5D%28%5BH%5D%29%28C%28%5BH%5D%29%28%5BH%5D%29C%28%5BH%5D%29%28%5BH%5D%29%5BC%40%40%5D3%28%5BH%5D%29%5BC%40%5D4%28%5BH%5D%29C%28%5BH%5D%29%28%5BH%5D%29C%28%5BH%5D%29%28%5BH%5D%29%5BC%40%5D%28%5BH%5D%29%28C%28%3DO%29C%28%5BH%5D%29%28%5BH%5D%29n5nc%28%5BH%5D%29c%28C%23N%29c5%5BH%5D%29%5BC%40%40%5D4%28C%28%5BH%5D%29%28%5BH%5D%29%5BH%5D%29C%28%5BH%5D%29%28%5BH%5D%29C%28%5BH%5D%29%28%5BH%5D%29%5BC%40%5D23%5BH%5D%29C1%28%5BH%5D%29%5BH%5D+&action=A) | 0.6401 |
| Carcinogenicity (Three-class) | [Non-required](http://lmmd.ecust.edu.cn/admetsar1/predict/?smiles=%5BH%5DO%5BC%40%5D1%28C%28%5BH%5D%29%28%5BH%5D%29%5BH%5D%29C%28%5BH%5D%29%28%5BH%5D%29C%28%5BH%5D%29%28%5BH%5D%29%5BC%40%40%5D2%28%5BH%5D%29%5BC%40%5D%28%5BH%5D%29%28C%28%5BH%5D%29%28%5BH%5D%29C%28%5BH%5D%29%28%5BH%5D%29%5BC%40%40%5D3%28%5BH%5D%29%5BC%40%5D4%28%5BH%5D%29C%28%5BH%5D%29%28%5BH%5D%29C%28%5BH%5D%29%28%5BH%5D%29%5BC%40%5D%28%5BH%5D%29%28C%28%3DO%29C%28%5BH%5D%29%28%5BH%5D%29n5nc%28%5BH%5D%29c%28C%23N%29c5%5BH%5D%29%5BC%40%40%5D4%28C%28%5BH%5D%29%28%5BH%5D%29%5BH%5D%29C%28%5BH%5D%29%28%5BH%5D%29C%28%5BH%5D%29%28%5BH%5D%29%5BC%40%5D23%5BH%5D%29C1%28%5BH%5D%29%5BH%5D+&action=A) | 0.5816 |
